# Identifying antibiotic-resistant strains via cell sorting and elastic-light-scatter phenotyping

**DOI:** 10.1007/s00253-024-13232-0

**Published:** 2024-07-03

**Authors:** Sharath Narayana Iyengar, Brianna Dowden, Kathy Ragheb, Valery Patsekin, Bartek Rajwa, Euiwon Bae, J. Paul Robinson

**Affiliations:** 1 Department of Basic Medical Sciences, Purdue University, West Lafayette, IN 47907 USA; 2 Weldon School of Biomedical Engineering, Purdue University, West Lafayette, IN 47907 USA; 3 School of Mechanical Engineering, Purdue University, West Lafayette, IN 47907 USA; 4 Bindley Bioscience Center, Purdue University, West Lafayette, IN 47907 USA

**Keywords:** MRSA, AMR, Flow cytometry, Bacteria sorting, Elastic light scatter, Machine learning, Bigfoot

## Abstract

**Abstract:**

The proliferation and dissemination of antimicrobial-resistant bacteria is an increasingly global challenge and is attributed mainly to the excessive or improper use of antibiotics. Currently, the gold-standard phenotypic methodology for detecting resistant strains is agar plating, which is a time-consuming process that involves multiple subculturing steps. Genotypic analysis techniques are fast, but they require pure starting samples and cannot differentiate between viable and non-viable organisms. Thus, there is a need to develop a better method to identify and prevent the spread of antimicrobial resistance. This work presents a novel method for detecting and identifying antibiotic-resistant strains by combining a cell sorter for bacterial detection and an elastic-light-scattering method for bacterial classification. The cell sorter was equipped with safety mechanisms for handling pathogenic organisms and enabled precise placement of individual bacteria onto an agar plate. The patterning was performed on an antibiotic-gradient plate, where the growth of colonies in sections with high antibiotic concentrations confirmed the presence of a resistant strain. The antibiotic-gradient plate was also tested with an elastic-light-scattering device where each colony’s unique colony scatter pattern was recorded and classified using machine learning for rapid identification of bacteria. Sorting and patterning bacteria on an antibiotic-gradient plate using a cell sorter reduced the number of subculturing steps and allowed direct qualitative binary detection of resistant strains. Elastic-light-scattering technology is a rapid, label-free, and non-destructive method that permits instantaneous classification of pathogenic strains based on the unique bacterial colony scatter pattern.

**Key points:**

• *Individual bacteria cells are placed on gradient agar plates by a cell sorter*

• *Laser-light scatter patterns are used to recognize antibiotic-resistant organisms*

• *Scatter patterns formed by colonies correspond to AMR-associated phenotypes*

**Supplementary Information:**

The online version contains supplementary material available at 10.1007/s00253-024-13232-0.

## Introduction

### Antimicrobial resistance

Antimicrobial resistance (AMR) is a growing global threat. The World Health Organization (WHO) has listed AMR as one of the top 10 public challenges and has called for urgent multilateral actions (WHO 2021). The rise of antibiotic-resistant bacteria is widely recognized as one of the most critical challenges amid various biological threats, including the emergence of new viral, fungal, and bacterial strains. These resilient microbial organisms persistently evolve and progressively limit antibiotic efficacy as a dependable treatment regimen (WHO 2021). One of the main reasons for the development of AMR in bacteria is the overuse or misuse of antibiotics in primary-care settings and in livestock (Llor and Bjerrum 2014; Martin et al. 2015). The most common and dangerous antibiotic-resistant bacteria listed by WHO include carbapenem-resistant *Enterobacteriaceae* such as *Escherichia coli*, *Klebsiella*, methicillin-resistant *Staphylococcus aureus* (MRSA), and various antibiotic-resistant *Mycobacterium tuberculosis* strains (WHO 2021). One of the most dangerous strains is MRSA, which predominantly causes skin infections. However, when introduced into the body, it can instigate bacteremia, pneumonia, or infection at surgical sites (CDC 2019). MRSA can be community or hospital acquired and accounts for 20–80% of hospital-associated infections globally (Fomda et al. 2014; Gurung et al. 2020; Krishnamurthy et al. 2014; Otto 2010). MRSA has developed resistance to nearly all beta-lactam antibiotics, primarily owing to their low binding affinity to the altered transpeptidase penicillin-binding protein 2a (PBP2a) that is encoded by the *mecA* gene (Hartman and Tomasz 1984). The ability of MRSA to cause a broad range of infections and to adapt to different environments has led to increased hospitalization costs and mortality (Gurung et al. 2020; Lowy 1998, 2003).

In the context of identifying bacterial pathogens from patient samples, agar plating is currently regarded as the gold standard for phenotypic detection (Abdou Mohamed et al. 2021; Alipour et al. 2014; Gosiewski et al. 2014; Harbarth et al. 2011; Salam et al. 2023). This mainly involves several rounds of cultivation to increase the number of bacteria and to obtain pure cultures (CDC 2019; Vasala et al. 2020). These cultures undergo antibiotic susceptibility testing (AST) using methods such as disk diffusion, microbroth dilution, and gradient test (Gajic et al. 2022). The minimum inhibitor concentration (MIC) is determined using the Clinical and Laboratory Standards Institute (CLSI) or European Committee on Antimicrobial Susceptibility Testing (EUCAST) standards (Vasala et al. 2020), which requires 72 h to complete (Baltekin et al. 2017; Iyengar 2021; Vasala et al. 2020). In the event of a polymicrobial infection, an expanding and diverse bacterial population on agar plates has been observed to engage in competition among the various bacterial species, resulting in the suppression of growth for certain bacteria (Hibbing et al. 2010). For the purpose of screening antibiotic-resistant strains, Bryson and Szybalski (1952) proposed the concept of an antibiotic-gradient plate to achieve a distribution of antibiotic concentration on a single plate. This approach was further utilized by another study performed by Liu et al. (2011) to determine the AST of enrofloxacin. However, there are various bacterial species that have resistance to enrofloxacin, such as *E. coli* (Piras et al. 2015), *Streptococci* sp. (Lukkana et al. 2016), or *Pseudomona*s sp. (Li et al. 2021), which needs to be further identified. In the field of medical diagnosis, the employment of genotypic analysis techniques has proven to be an effective means of reducing the time of diagnosis. Real-time quantitative polymerase chain reaction (qPCR) is a preferred approach in this regard. It has been utilized in the recent COVID-19 pandemic owing to its ability to provide rapid results with high sensitivity and specificity (Tahamtan and Ardebili 2020). However, pure DNA extraction is needed for effective results, which depend on the starting sample type. PCR directly on clinical samples is confined to only a few sample types, such as sterile body fluids (Cherkaoui et al. 2010; Kotilainen et al. 1998; Trung et al. 2019; Xu et al. 2003). Standard PCR as a stand-alone technique cannot provide any information about the viability of bacteria and has low sensitivity and specificity if the starting sample is a complex body fluid such as blood (Cangelosi and Meschke 2014; Dietvorst et al. 2022; Narayana Iyengar et al. 2021; Sinha et al. 2018; Trung et al. 2019). In addition, in some instances, genotype does not correspond to a phenotype, which makes it more challenging to treat the disease (Kunze et al. 2015; Yoon and Jeong 2021).

MALDI-TOF is another commonly used method for detecting bacteria and can provide results in minutes. However, MALDI-TOF has limitations in differentiating closely related bacteria species owing to a limited bacterial protein database, which leads to incorrect identification (Rychert 2019). Other techniques, such as fluorescent in-situ hybridization (FISH), are rapid, easy to use, and highly specific to bacterial biomarkers, but the antibodies specific to all the bacterial strains are not available (Singhal et al. 2015). Furthermore, microfluidic devices have also gained more interest in recent years, as they have the capability to detect bacteria from a small starting sample volume. However, they are still limited in terms of having a tradeoff between high throughput and separation efficiency (Iyengar et al. 2021; Kumar et al. 2021), and sensitivity based on starting sample type, and there is a need for developing new fabrication techniques for cheap mass production of chips (Zhang et al. 2018). Therefore, there is an immediate necessity to create a technique that can detect and recognize AMR occurrence rapidly, economically, and efficiently to ensure that the patient receives the appropriate treatment.

Flow cytometry instruments are extensively used in the field of microbiology due to their ability to analyze and sort single cells in real time. These tools are capable of distinguishing between live and dead cells and can be coupled with any downstream analysis, thereby providing valuable insights into complex microbial communities (Davey and Kell 1996; Robinson et al. 2023; Shapiro 2003; Steen 1990). For microbiological applications in flow cytometers, forward-scatter (FSC) and side-scatter (SSC) signals can be combined with fluorescence staining (such as specific antigens or nucleic-acid dyes or stains for enzyme regulation, etc.) to sort them by gating on density plots (FSC vs. SSC) (Davey and Kell 1996; Robinson et al. 2023; Shapiro 2003; Steen 1990; Veal et al. 2000). However, the staining methodology is limited by the number of lasers and detectors available in the flow cytometers. The emission spectra of many stains exhibit similarities that can result in undesired signal “spillover” across different detectors, even when optical filters are employed with great care. Signal compensation is a commonly employed technique that accounts for spectral overlap effects and allows for separating abundance information from individual labels (Bagwell and Adams 1993). However, when the emission peaks from multiple stains overlap and are accompanied by autofluorescence from organisms and media, the task becomes increasingly difficult (Roederer 2001). These limitations motivated the development of spectral flow cytometry (Grégori et al. 2012, 2014; Robinson 2004; Robinson et al. 2004). The use of flow cytometers for conducting experiments with pathogenic microorganisms poses a significant challenge owing to the inherent biosafety concerns. The instrument is typically operated inside a biosafety hood to ensure user safety; however, not all flow cytometers are compatible with this requirement (Kennedy et al. 2011; Kennedy and Wilkinson 2017).

Here, we present a rapid, label-free, and non-destructive phenotypic approach by combining the ThermoFisher Bigfoot spectral sorter with elastic light scattering (ELS) technology for the detection and identification of viable bacteria. To showcase the potential of our method in identifying antimicrobial resistance in *Staphylococcus aureus* strains, non-pathogenic bacteria were utilized as a proof of concept. Our approach effectively detects and identifies both resistant and non-resistant strains, offering a promising solution for combating the growing issue of AMR.

**Fig. 1:**
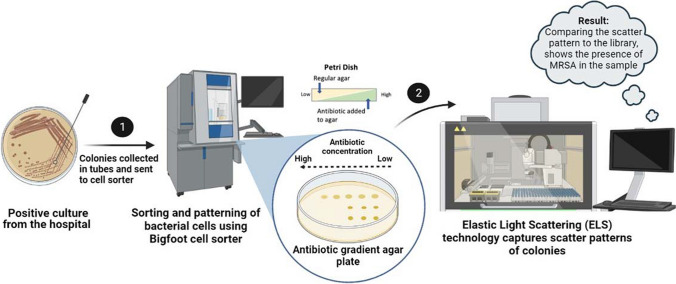
The schematic shows an overview of the proposed methodology for detecting and identifying resistant strains. Bacterial colonies from positive culture are inoculated into media tubes and introduced into the Bigfoot cell sorter. Single bacterial cells are sorted and patterned on an antibiotic gradient plate (AGA). Growth of bacteria in the high-concentration region of the AGA shows the presence of an antibiotic-resistant strain. The AGA plate is introduced into the ELS instrument to identify the resistant strain by comparing the recorded colony scatter pattern to the existing scatter patterns in the library

The schematic in Fig. 1 summarizes the steps involved in the proposed method. In step 1, a bacterial colony is inoculated into a buffer or media from the positive culture and directly introduced into the cell sorter. Single bacteria are sorted (by gating on the density plot), patterned at predesignated spots onto an antibiotic gradient agar plate (AGA), and incubated at 37 °C for several hours. An AGA plate has a gradient of antibiotic concentration across the plate. The presence of antibiotic-resistant bacteria can be observed by the growth of colonies at the high antibiotic–concentration region on the plate. In step 2, elastic scatter patterns produced by the bacterial colonies grown on the AGA plate are acquired. The recorded scatter patterns are compared to the known ones in the ELS library using pattern-recognition and machine-learning techniques. Based on the percentage of scatter-pattern similarities, the unknown bacterial colonies are classified. This proposed methodology is promising for detecting pathogenic organisms from a variety of biological samples, such as blood, urine, etc., for infection diagnosis. It can also be extended to other samples, such as food and water, for detecting contamination.

### Thermo Fisher Bigfoot flow sorter

**Fig. 2:**
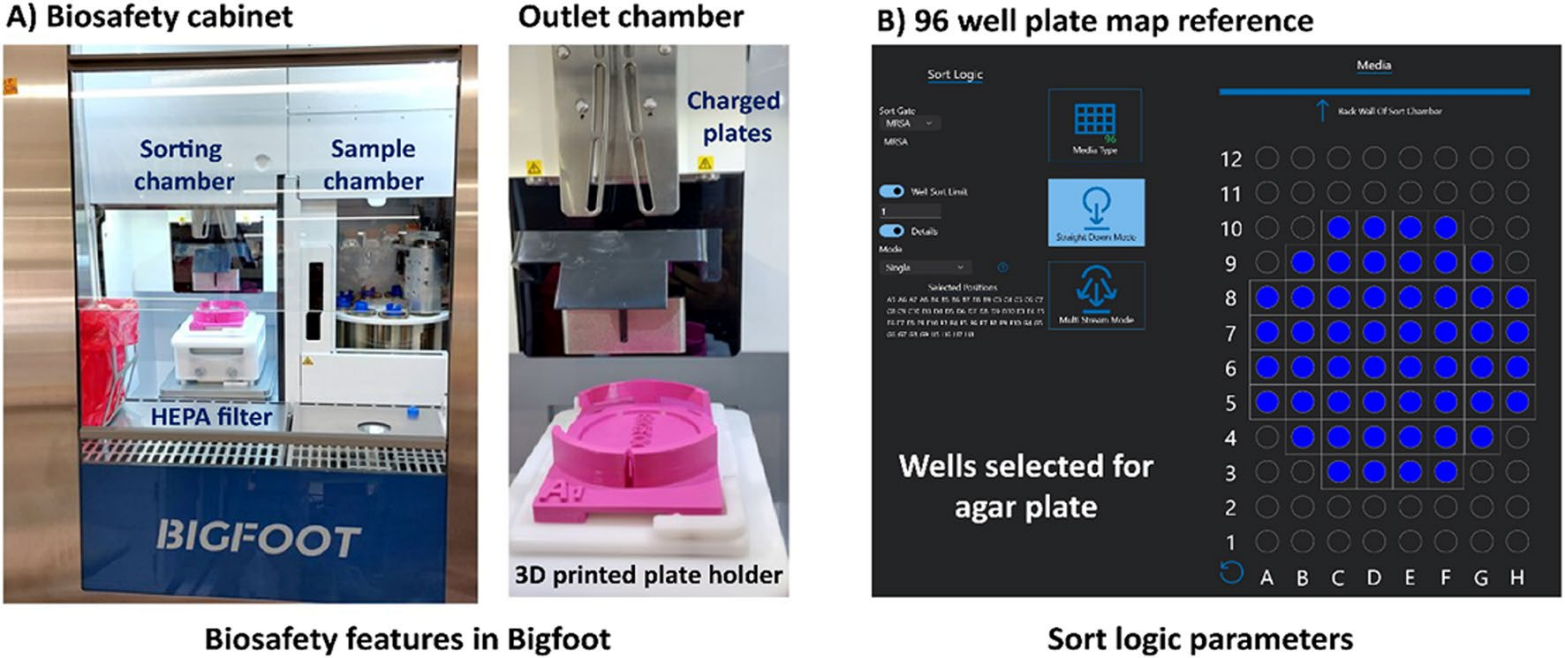
Bigfoot biosafety features and sorting parameters. **A** The biosafety cabinet with three levels of biosafety features is highlighted. The features include 1. biocontamination chamber with HEPA filters for purified air circulation, 2. separate chamber for handling pathogenic samples, which limits access to users, and 3. separate chamber for sorting, which provides safety from splashing. **B** Screenshot from Bigfoot software showing a 96-well plate reference map with different sort parameters used for patterning single organisms onto an agar plate

The Bigfoot spectral cell sorter (ThermoFisher Scientific Inc., Waltham, MA) has been designed to rapidly and accurately sort and isolate cells based on their physical and fluorescent properties. It can sort up to 70,000 cells per second and offers a high degree of customization, with the potential for up to 9 lasers and 60 individual detectors (ThermoFisher 2020). It was designed to aseptically isolate and purify cells for downstream applications such as gene-expression analysis, single-cell sequencing, and cell-based assays (ThermoFisher 2020). Bigfoot is a spectral cytometry instrument; therefore, it can sort cells based on their spectral signatures. Real-time spectral unmixing can be performed to enable sorting cells for further downstream analysis. In addition, Bigfoot can handle pathogenic organisms because of its special biosafety features (ThermoFisher 2020). Figure 2A (left) shows the biosafety cabinet in the Bigfoot. Bigfoot has three levels of biosafety protection. First, a biosafety chamber with HEPA filters, similar to a biosafety level class 2 hood for biocontainment and aerosol management. Second, the sample is placed in a separate chamber, preventing direct user interaction. Third, the outlet for sorting is also located in a separate chamber with a door that prevents splashing into the surroundings and protects the users.

The voltage plates that sort cells based on their charge are shown in Fig. 2A (bottom right). In this communication, our goal is to report the development of a label-free method for patterning bacteria onto an agar plate.

## Materials and methods

### Preparation of agar for bacterial plating

Four different media were used to culture bacteria, with tryptic soy agar being the primary choice owing to its non-selective nature. In this study, we utilized xylose lysine tergitol-4 (XLT4) base agar for the selective growth of *Salmonella* sp. and violet red bile glucose (VRGB) agar for the selective growth of *Enterobacteriaceae* spp. These media have been specifically formulated to facilitate the growth of the target microorganisms while suppressing the growth of undesired ones. VRGB agar was prepared by dissolving 38.5 g of agar in 1 L of water. For preparing XLT4 agar, an XLT4 agar supplement containing sodium tetradecyl sulfate solution (26–28%) was used to enhance the growth of *Salmonella* sp. XLT4 (4.6 mL) supplement was added to 59 g of 1 L XLT4 base agar. All agar suspensions were autoclaved and cooled before pouring onto an empty agar plate.

### Preparation of layered oxacillin AGA plates for MSSA and MRSA

AGA plates contain two layers of TSA agar. The first layer contains a defined concentration of oxacillin, while the second layer is TSA agar without oxacillin. The setup used for preparing the AGA plate is shown in Supplementary Fig. S2. To obtain the two agar layers, agar solutions were prepared as follows: For the first agar layer, three different antibiotic concentrations (1, 2, and 4 μg/mL) were tested for our experiments. To obtain these concentrations, a stock oxacillin solution (64 μg/mL) was prepared by dissolving 16 mg oxacillin sodium salt, 95%, in 250 mL distilled water. A dilution series was performed using distilled water to obtain 1, 2, and 4 μg/mL oxacillin from the stock solution. These working concentrations were chosen based on the CLSI and CDC reports showing that the breakpoint oxacillin concentration for MRSA is ≥ 4 ug/mL (CDC 2019; CLSI 2020). For example, to obtain 500 mL of 4-μg/mL oxacillin TSA agar suspension (first layer of AGA agar), 20 g TSA agar powder was added to 250 mL of 8-μg/mL oxacillin and was made up to 500 mL using distilled water. For the second layer, 40 g TSA agar powder was added to 1 L of distilled water (without oxacillin). Agar suspensions with and without oxacillin were autoclaved. After the agar suspension cooled to around 50 °C, 20 mL of the first agar suspension (containing agar and oxacillin mixture) was poured onto the empty agar plate (100 × 15 mm) and placed in the slanted position. This forms the first layer of agar. Once this first layer solidifies, 20 mL of the second agar suspension was poured on the first layer after placing the plate in the horizontal position. The second layer was allowed to solidify. This resulted in an antibiotic gradient across the plate with a very high concentration of oxacillin at one end of the plate and essentially zero concentration on the other. After preparation, the AGA plates were stored in a 4 °C refrigerated room for at least 17 h before starting the experiments.

### Preparation of bacteria samples

*Escherichia coli* ATCC 25922, methicillin*-*susceptible *Staphylococcus aureus* ATCC 23235, *Salmonella enteritis* ATCC 13076, and *Klebsiella pneumonia* ATCC 13883 ordered from ATCC were used for experiments. Methicillin-resistant *Staphylococcus aureus* PU54 REAR-16 (de-identified human clinical isolate), which is resistant to oxacillin and methicillin antibiotics, was used for our experiments. Before starting the experiments related to bacteria, all bacteria were grown overnight at 37 °C in the Fisher Scientific Isotemp incubator on a TSA agar plate. *Salmonella enteritis was* also grown on XLT4 agar, which is a selective medium for *Salmonella* sp. From the overnight culture, colonies from each bacterium were inoculated into phosphate buffer solution (PBS), and the concentration was adjusted to ${10}^{6}$ CFU/mL by measuring the initial optical density (OD) using a spectrophotometer (WPA Biowave CO8000 cell density meter).

### Bigfoot sorting parameters for bacterial patterning

As discussed in Fig. 2, up to 52 wells from the 96-well plate could be mapped onto the agar region using the standard 96-well pattern provided in the cytometer software. One milliliter of 10^6^ CFU/mL bacteria in PBS solution was used for sorting and patterning. Sorting parameters set in the control software were as follows (see Fig. 2B): 1. *media type:* 96-well plate, 2. *well sort limit* = 1 (indicating that a single bacterial cell will be deposited at a spot), 3. *mode*: single down mode (this mode option allows more precise deposition).

### Waste and contamination management

When handling bacteria, it is crucial to maintain an aseptic environment and dispose of waste correctly, especially if the bacteria in question are pathogenic or antibiotic resistant. After conducting experiments with bacteria, the Bigfoot sorter system undergoes a four-stage manual washing process to guarantee thorough cleaning. The steps are as follows. Step 1: 10% bleach (Clorox) for 5 min. Step 2: Coulter Clenz cleaning agent (Beckman-Coulter) for 5 min. Step 3: 70% ethanol for 10 min. Step 4. Hellmanex solution for 5 min. Upon completion, a clean water sample is analyzed to determine if any particles were detected by the instrument. If no particles were found, the washing step was considered successful. However, if any particles were detected, the washing step is repeated to ensure complete cleanliness. After the final shutdown, the sorter undergoes an automated washing process again. Following completion of the shutdown, the waste accumulated in the waste chamber is subjected to further treatment with 10% bleach for a minimum of 4 h prior to disposal.

### Elastic light scattering technology

The ELS colony-analysis technique for the rapid identification of bacteria was first proposed by researchers at Purdue University (Banada et al. 2009; Bayraktar et al. 2006). The method was founded upon the distinctive scatter patterns of bacterial colonies, which are correlated with their morphology. Initially, the technique was employed to identify foodborne pathogens (Bae et al. 2010, 2012b; Buzalewicz et al. 2019; Huff et al. 2012; Marcoux et al. 2014; Pan et al. 2015; Rajwa et al. 2010; Singh et al. 2014, 2015).

**Fig. 3:**
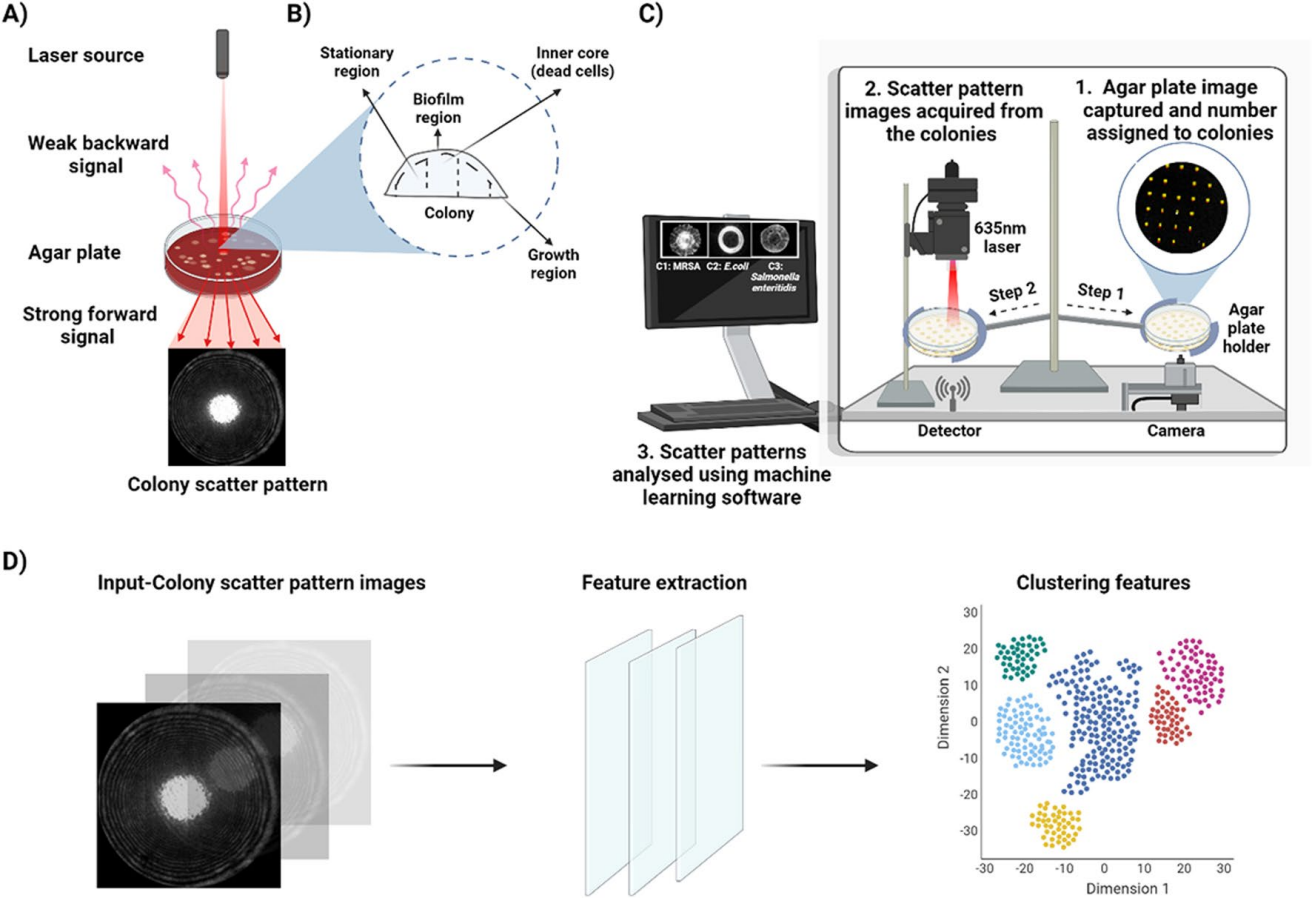
Schematic showing the steps involved in recording and processing the bacterial colony scatter patterns using ELS technology and machine learning. **A** The working principle of ELS is briefly described. **B** Bacterial colony composition showing variation in cell growth such as cells in stationary phase, growth phase, dead cells, and biofilm region. These morphological characteristics are captured in the form of a unique colony scatter pattern using ELS. **C** The schematic of the ELS device highlights the different components of the device and the steps involved in acquiring scatter patterns. **D** Image processing using machine-learning software for identification and classification of bacteria based on the extracted features

The schematic in Fig. 3 shows the working principle of ELS technology (Fig. 3A), the ELS instrument (Fig. 3B), and the scatter pattern–image feature extraction for bacterial identification (Fig. 3C). The ELS device includes a laser diode (635-nm wavelength and 1-mm beam diameter) that is used to generate the scatter patterns of the inspected bacterial colony, an agar-plate holder, an LED backlight below the agar-plate holder for illumination, and a camera above the agar-plate holder to capture the images of the bacterial colonies.

The operation of the system has been described elsewhere (Bae et al. 2011, 2012a, 2012b). Briefly, the laser light irradiates microbial colonies grown on the agar plate. The light is scattered by a colony (acting as a highly deformed, imperfect lens), forming a strong forward- and weak backward-scatter signal. The weak backward scatter is ignored, and the forward-scattered light is captured by a camera. The resulting complex scatter pattern is largely influenced by the internal heterogeneity of the microbial colony (Fig. 3C). The scatter pattern may vary depending on factors such as the bacterial species’ colony-formation ability, the growth medium type, humidity, and response to antibiotics. The scatter pattern of each colony is visualized in real time and recorded in a database. Later on, computer-vision and machine-learning techniques are utilized to extract features from the pattern images. These features are then used in a supervised learning model for pattern classification, revealing the bacterial identity (Fig. 3D).

Extracting features from the images and establishing a quantitative description of the ELS patterns is the most crucial part of the data-processing step (Liu et al. 2007). Some of the most common features that can be extracted from the images and spatial patterns include shape, edge descriptors, texture, and color (Liu et al. 2007). Many studies have previously reported unique colony scatter patterns formed by different bacterial species and strains (Alsulami et al. 2018; Banada et al. 2009; Bayraktar et al. 2006; Singh et al. 2014; Tang et al. 2014). The ELS technique relies primarily on Zernike and pseudo-Zernike moments (Flusser et al. 2016; Liu et al. 2007; Teague 1980), which offer the advantage of having transitional and rotational invariance and high efficiency (low redundancy) (Khotanzad and Hong 1990; Kim and Kim 2000; Liu et al. 2007; Teh and Chin 1988).

The computation of Zernike (and pseudo-Zernike) polynomials and moments has been described elsewhere in detail (Flusser et al. 2016; Irvine et al. 2022; Mukundan and Ramakrishnan 1995). Briefly, in order to derive Zernike moments, the first step is to project the image function onto the Zernike basis functions. These basis functions are mathematically defined and specifically engineered to capture the spatial frequency content of the image:

$${V}_{n,m}(x,y)={R}_{n,m}(\rho ){e}^{jm\phi }$$

where *n* and *m* are nonnegative integers with *n* ≥ *m* ≥ 0, $\rho$ is the radial distance (the distance from the origin to a point on the unit disk), $\Phi$ is the azimuthal angle (the angle between the positive *x*-axis and the line connecting the origin and the point), and *R*_*n,m*_(*ρ*) are the radial polynomials defined as

$${R}_{n,m}(\rho )=\sum_{k=0}^{(n-m)/2} \frac{(-1{)}^{k}(n-k)!}{k!((n+m)/2-k)!((n-m)/2-k)!}{\rho }^{n-2k}$$

The Zernike moment of order *n* and repetition *m* for an image function *f*(*x*,*y*) is then defined as

$${A}_{n,m}=\frac{n+1}{\pi }{\int }_{0}^{2\pi } {\int }_{0}^{1} f(\rho ,\phi ){V}_{n,m}^{*}(\rho ,\phi )\rho d\rho d\phi$$

where ${V}_{n,m}^{*}$ is the complex conjugate of ${V}_{n,m}$.

The larger the degree of the polynomial, the larger the feature vector (Bayraktar et al. 2006; Flusser et al. 2016; Irvine et al. 2022).

Another form of Zernike moments, called pseudo-Zernike, has also been used by ELS as they are superior in terms of feature-representation capabilities, reduced noise, and better feature extraction for complex features compared to Zernike moments (Chong et al. 2003; Teh and Chin 1988).

The pseudo-Zernike basis function is defined as

$${V}_{n,m}(x,y)=(n+1){R}_{n,m}(\rho ){e}^{jm\phi }$$

where *R*_*n,m*_(*ρ*) are the radial polynomials:

$${R}_{n,m}(\rho )=\sum_{k=0}^{(n-|m|)} \frac{(-1{)}^{k}(n-k)!}{k!((n+|m|)/2-k)!((n-|m|)/2-k)!}{\rho }^{n-2k}$$

Finally, the pseudo-Zernike moment of order *n* and repetition *m* is defined as

$${A}_{n,m}=\frac{1}{\pi }{\int }_{0}^{2\pi } {\int }_{0}^{1} f(\rho ,\phi ){V}_{n,m}^{*}(\rho ,\phi )\rho d\rho d\phi$$

In this work, we mainly used pseudo-Zernike moments for extracting the features from the colony scatter patterns for bacterial identification and classification. For the classification purposed, we utilized a common support vector machine technique (SVM) with implementation details that have been previously showcased and discussed in multiple previous publications, including Bae et al. (2016), Bae et al. (2012a), Bae et al. (2012b), Banada et al. (2007), Banada et al. (2009), Huff et al. (2012), Huff et al. (2006) and Singh et al. (2014).

### Principal component analysis (PCA)

Principal component analysis (PCA) has been extensively described in the literature (Jolliffe and Cadima 2016). Briefly, PCA is a statistical method for dimensionality reduction whereby complex, high-dimensional data are transformed into a lower-dimensional space defined by principal components (PCs). This transformation facilitates the representation of complex data features in a simpler, more understandable format (Jolliffe and Cadima 2016; Lever et al. 2017; Migenda et al. 2024). The first principal component (PC1) is determined in such a way that it maximizes the variance of the data projected onto it, effectively minimizing the distance between each original data point and its projection onto PC1 (Lever et al. 2017). The second principal component (PC2) is computed to be orthogonal to PC1, capturing the second highest variance, with subsequent PCs being geometrically orthogonal to all preceding ones and capturing progressively smaller amounts of variance (Lever et al. 2017). The first few PCs generally represent the most significant variance within the data, while later PCs tend to reflect residual noise (Yeung and Ruzzo 2001). The orientation of the principal components is described by eigenvectors, which indicate the direction of maximum variance, whereas the magnitude of variance accounted for by each PC is quantified by its corresponding eigenvalue (Jolliffe and Cadima 2016). PCA plots, which graphically represent the PCs, are utilized to identify clusters of data points with similar characteristics, thereby facilitating the visualization of data points grouping (Lever et al. 2017; Yeung and Ruzzo 2001).

## Results

### Single bacterial cell patterning using the cell sorter

**Fig. 4:**
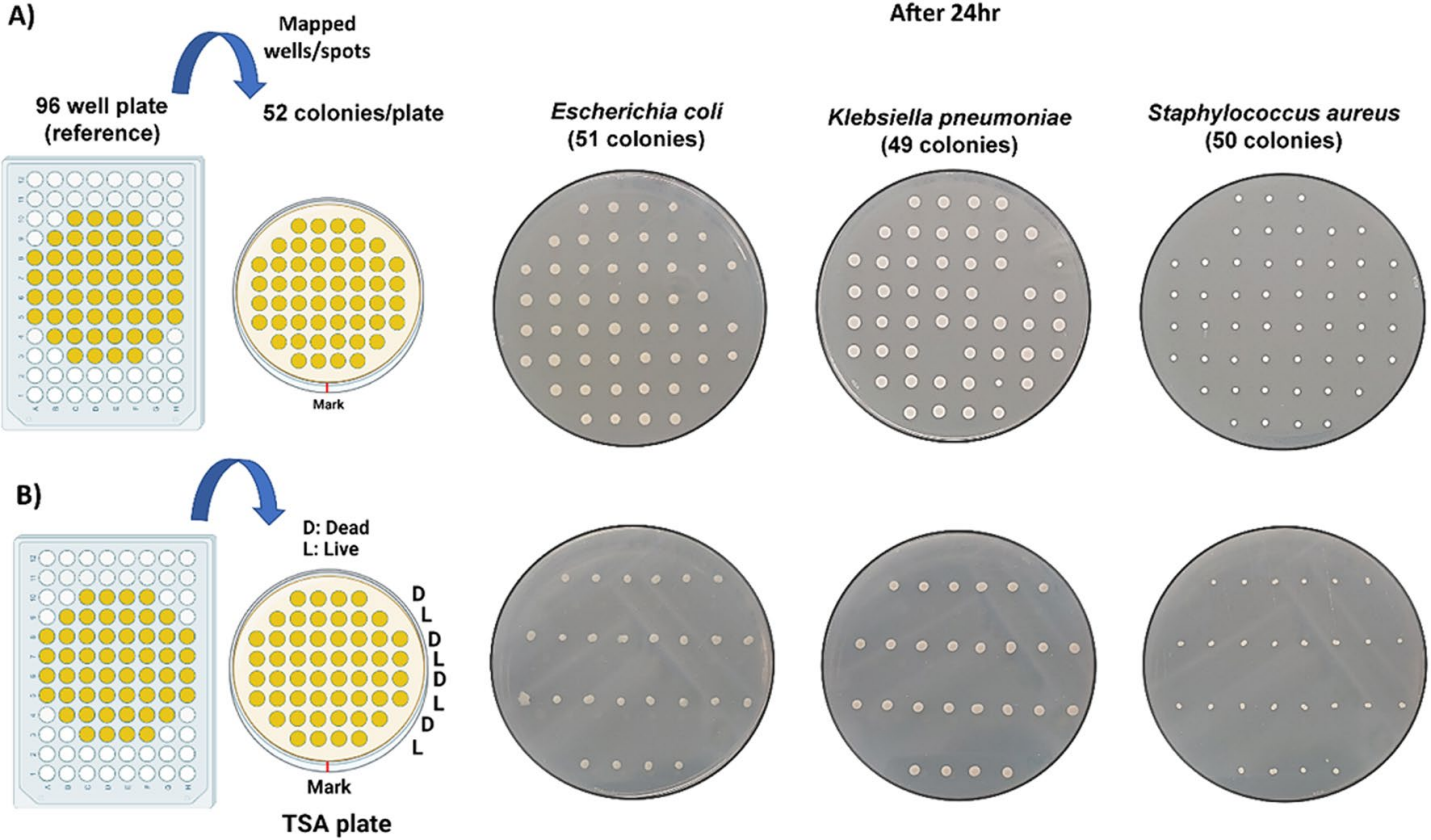
Single bacterium cell patterning using the Bigfoot cell sorter. **A** Ninety-six-well plate used as a reference for mapping and depositing cells at desired spots onto an agar plate. Single bacterium of three different species (*E. coli*, *K. pneumonia*, and *S. aureus*) patterned and plated on TSA agar plate. **B** SYTO9- and PI-stained live and heat-killed bacteria were patterned in alternate rows on a TSA agar for all three bacterial species in separate experiments

As proof of principle, we initially optimized the system for cell sorting and patterning using three different bacterial species, *E. coli K12*, *Klebsiella pneumoniae*, and *Staphylococcus aureus*, patterned on TSA agar plate in separate experiments as shown in Fig. 4A. Images of the agar plates were captured after 24 h incubation at 37 °C. The event rate for all three bacterial experiments was 1000 cells/s at a bacterial concentration of 10^6^ CFU/mL. Patterning of cells on the agar plate takes about 13 s; this time may vary depending on the concentration of bacterial cells in the sample and the event rate. The probability of depositing a bacterial cell at a desired spot ranged from 98% (for *E. coli* with 51 colonies), 94% (for *Klebsiella pneumoniae* with 49 colonies), to 100% (*Staphylococcus aureus* with 52 colonies). The probability percentage was calculated based on the total number of colonies counted per plate divided by the desired number of colonies per plate (52) multiplied by 100.

In another experiment, we patterned live and heat-killed bacterial cells in alternate rows for all three bacterial species on TSA agar, as shown in Fig. 4B. Live and dead bacterial cells were gated based on staining with SYTO9G (for live cells) and propidium Iodide (for dead cells). As expected, viable bacteria stained with SYTO9G were observed in all plates after 24 h of incubation. This demonstrates the unique ability of the Bigfoot cell sorter to precisely deposit one cell at the region of interest along with sorting live from dead cells in a given sample. This cannot be achieved using the conventional streak plate method, where it is not possible to control the growth of bacterial colonies at the desired spot, and multiple dilutions are needed to obtain single, separated colonies on a plate.

### Patterning methicillin-susceptible and -resistant S. aureus strains on antibiotic gradient plate

Oxacillin is a standard antibiotic used to determine the minimum inhibitory concentration (MIC) of MSSA and MRSA for antibiotic susceptibility testing. Calculation of MIC for both MSSA and MRSA by standard microdilution method on a 96-well plate is shown in Supplementary Fig. S1. An AGA has a gradient of antibiotic concentrations with zero concentration at one end of the plate and an exceedingly high concentration at the other. This is achieved by creating two layers of agar, as described in the “Materials and methods” section. Four different oxacillin (oxa) concentrations were prepared: 1, 2, 4, and 16 μg/mL (*n* = 3, for each case). These concentration values correspond to the highest oxa concentration on the plate. MSSA and MRSA were patterned using the cell sorter on TSA agar and were used as positive controls (*n* = 3).

**Fig. 5:**
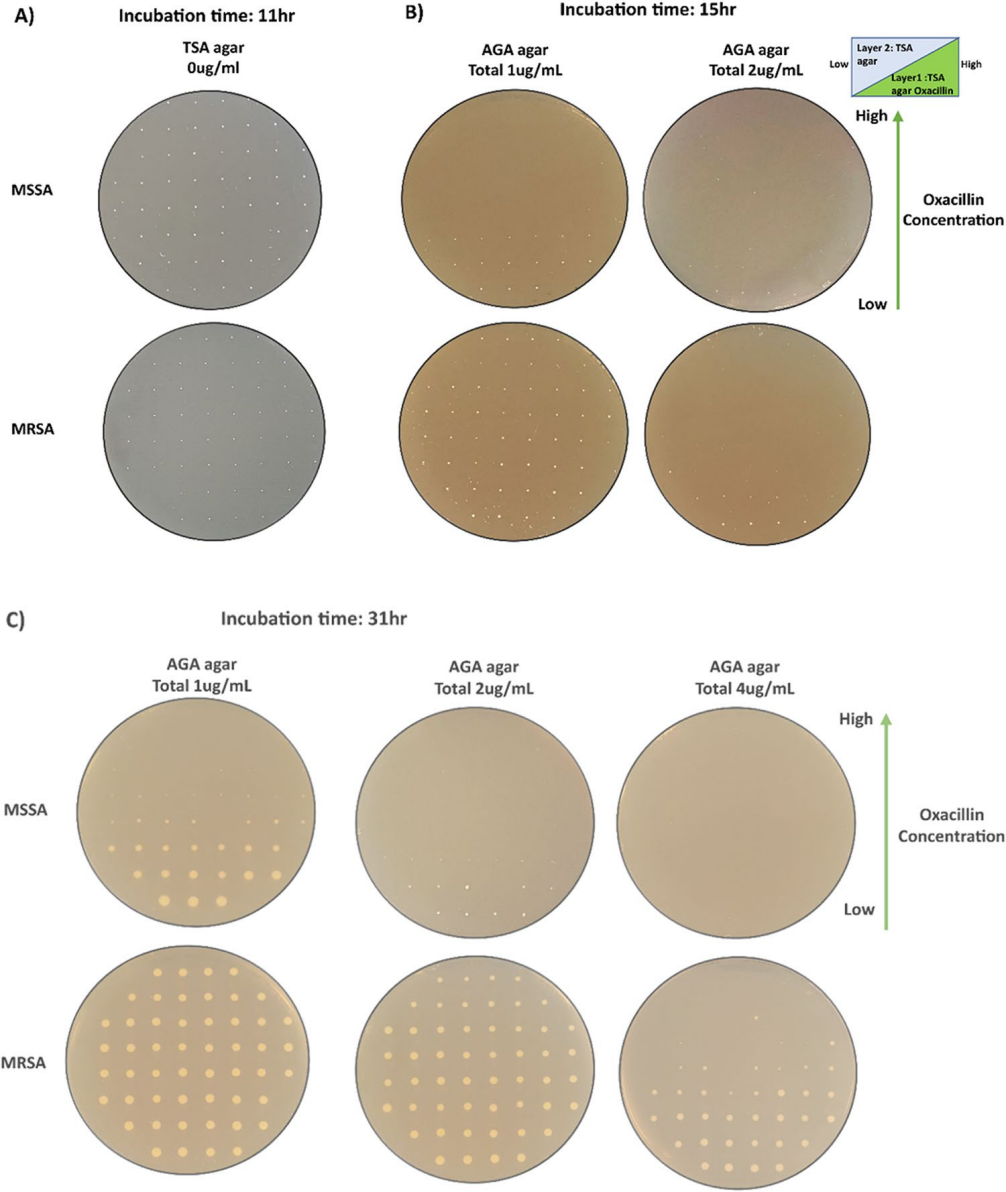
Patterning of single methicillin-susceptible *Staphylococcus aureus* (MSSA) and methicillin-resistant *Staphylococcus aureus* (MRSA) on an AGA plate. **A** MSSA and MRSA colonies patterned on TSA (as positive controls) after 11 h. **B** MSSA and MRSA were patterned on 1- and 2-μg/mL oxacillin AGA plates. After 15 h of incubation, growths of MSSA at low oxacillin (oxa) concentrations were observed for 1- and 2-μg/mL plates. However, no MSSA growth was observed at any region on 4-μg/mL AGA plates. On the other hand, MRSA growth was observed across both low and high oxa concentrations for 1-μg/mL AGA plates. MRSA grew only at low oxa concentrations regions in 2-μg/mL plates after 15 h. No MRSA growth was observed on 4-μg/mL AGA plates. **C** After incubating for 31 h, MSSA growth was observed in low oxa concentration regions of 1- and 2-μg/mL AGA plates. A few small colony variants (SCVs) of MSSA were observed close to high oxa-concentration regions on 1 μg/mL AGA plates. No growth of MSSA was observed in 4-μg/mL plates. MRSA colonies were observed across all regions of 1- and 2-μg/mL AGA plates. In addition, some colony formation in low-to-intermediate oxa concentration regions was observed on 4-μg/mL plates after 31 h of incubation

The plates were incubated at 37 °C overnight. One sample set is shown for each oxa concentration in Fig. 5. Panel A shows the growth of MSSA and MRSA on TSA agar (positive control) after 11-h incubation. On the other hand, growth of MSSA was observed after 15 h in the low oxacillin concentration regions of 1- and 2-μg/mL AGA plates, respectively (Fig. 5B). Initial colony formations could already be observed at around 11 to 12 h (not shown). No growth of MSSA was observed for 4 and 16 μg/mL AGA plates after 15 h (see Supplementary Fig. S3). In contrast, MRSA growth was observed for all regions on a 1-μg/mL AGA plate and at low oxacillin concentration regions of a 2-μg/mL AGA plate after 15 h.

All plates were incubated for an extended period to allow for colony growth. Figure 5C shows the growth of both MSSA and MRSA after 31-h incubation on 1-, 2-, and 4-μg/mL AGA plates. Small colonies of MSSA were observed closer to the high oxa concentration regions in a 1-μg/mL AGA plate, whereas MSSA was observed only at low oxa concentration regions in a 2-μg/mL AGA plate. MRSA growth was observed across the plate in 1- and 2-μg/mL AGA plates (Fig. 5C). Interestingly, MRSA growth was also observed in the low to intermediate oxa concentration regions on the 4-μg/mL AGA plate after 31-h incubation (Fig. 5C), which was not observed at 15 h (in Fig. 5B).

The colonies formed on AGA plates indicate that in the presence of antibiotics both MSSA and MRSA exhibit slower growth rates and form smaller colonies near regions with high oxa concentrations. This is due to the stress from antibiotics and demonstrates that MRSA can be easily identified by looking for growth at high oxa concentration in a 2-μg/mL AGA plate or at low- to intermediate-concentration regions on a 4-μg/mL AGA plate.

### Scatter patterns of different bacteria using elastic light scattering

**Fig. 6:**
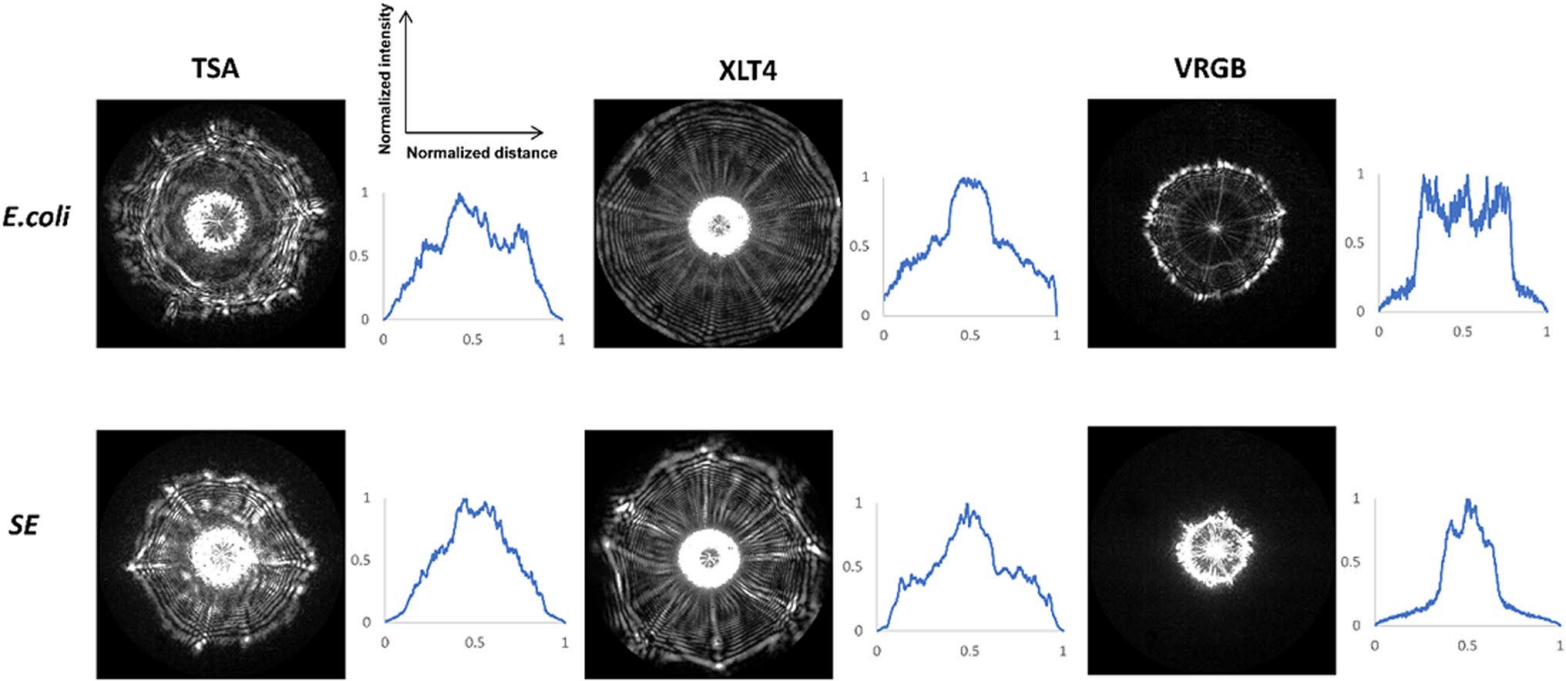
Unique scatter patterns of two different bacteria grown on three different agars; TSA, XLT4, and VRGB. Colonies were sorted using the Bigfoot cell sorter and incubated for 11 h. Colony scatter patterns were captured using the ELS device. Scatter patterns of *E. coli* and *Salmonella enteritidis* (SE) are shown. Unique scatter patterns differ based on the agar selected. ELS technology can be used for bacterial identification and classification

Initial experiments were performed to capture the scatter patterns of two different bacterial species using the ELS approach. Two different bacteria were patterned on three different agars (tryptic soya agar (TSA), xylose lysine tergitol-4 (XLT4), and violet red bile glucose (VRGB) agar) using the cell sorter and incubated for 11 h at 37 °C. Both XLT4 and VRGB agars are selective agars that permit the growth of specific types of bacteria. More information about these agars can be found in the “Material and methods” section. In Fig. 6, scatter patterns of two different bacteria, *E. coli* K12 and *Salmonella enteritidis* (SE), are compared. Their corresponding intensity profiles were obtained using ImageJ 1.53k software. It can be observed that the scatter patterns of the same bacteria differ when they are grown on different agar plates. *E. coli* and SE have distinctive scatter patterns that can be utilized for bacterial identification. The scatter pattern features observed directly reflect the unique morphological characteristics and growth behavior of each bacterial species.

### Comparison of phenotypic characteristics between antibiotic-susceptible and -resistant S. aureus using colony scatter patterns

We have shown that only MRSA colonies grow in high concentrations of oxa, specifically at 2-μg/mL, and in low-to-intermediate concentrations of 4 μg/mL on AGA plates (Fig. 5). This is the first step for demonstrating the presence of a resistant strain in the sample. For samples containing unidentified microbial species, the identification of bacterial species is critical for determining the appropriate treatment method.

**Fig. 7:**
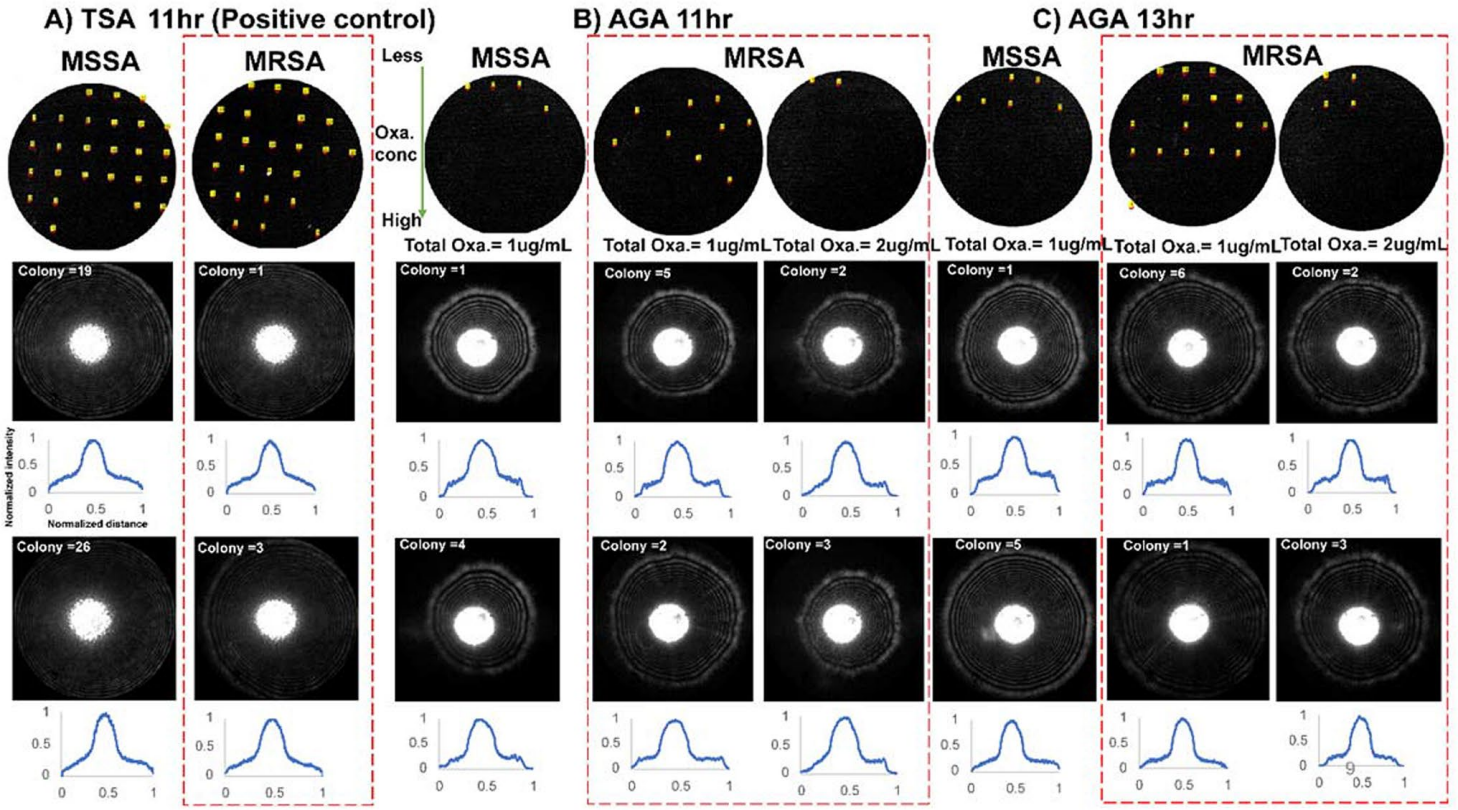
Comparison of colony scatter patterns of MSSA and MRSA grown on TSA and AGA plates. The colony images from ELS with assigned colony numbers and the normalized intensity vs distance plots are shown. **A** The colony scatter patterns of three random colonies of MSSA and MRSA after 11 h incubation on the TSA plate. MSSA and MRSA demonstrated similar colony scatter patterns on TSA. **B** MSSA on 1-μg/mL and MRSA on 1- and 2-μg/mL oxa AGA plates after 11 h incubation. Scatter patterns for both MSSA and MRSA differed on AGA plates compared to TSA. **C** Scatter patterns of MSSA and MRSA after 13 h incubation. Heterogeneity in scatter patterns was observed for MSSA and MRSA colonies based on the concentration of antibiotics on the AGA plates

To explore the method’s ability to differentiate the resistant from the non-resistant strain, MSSA and MRSA were patterned on AGA plates using the cell sorter. The scatter patterns were captured using the ELS system (Fig. 7). MSSA and MRSA grown on TSA agar were used as positive controls (Fig. 7A). One-μg/mL and 2-μg/mL oxa AGA plates were used to compare the scatter patterns of MSSA and MRSA after 11- and 13-h incubation (Fig. 7B and 7C). Although the colonies formed at 11 and 13 h were too small to be observed by the naked eye, the ELS system could recognize them. AGA plates with 4-μg/mL oxa were not used, since no growth of MSSA can be observed in that condition (see Fig. 5).

MSSA colonies were not identified by ELS on 2-μg/mL AGA plates at 11 and 13 h, so the scatter patterns of MSSA are shown only for the 1-μg/mL case. During its operation, the ELS system software captures the image of the agar plate, and a number is assigned for each observed colony. In the next step, the system scans the plate to capture the colony scatter patterns. Among the results shown, examples of scatter patterns from two representative colonies per plate for MSSA and MRSA on TSA and AGA plates are highlighted along with their respective normalized intensity and distance plots. The red dashed box highlights the scatter patterns of MRSA colonies. The concentric rings observed in the scatter pattern are a unique signature for *Staphylococcus aureus* species and occur mainly owing to their growth along the colony height (growth in vertical direction). These observations have been reported in previous studies (Alsulami et al. 2018). Both MSSA (colonies 1, 19, and 26) and MRSA (colonies 1, 19, and 3) grown for 11 h on a TSA agar plate (Fig. 7A) had similar colony scatter patterns, and no observable differences were found.

As shown in Fig. 5, there is a significant variation in the colony size for both MSSA and MRSA based on the concentration of oxa on the AGA plate. This was also confirmed by observing similar normalized intensity plots for both MSSA and MRSA. In general, the scatter patterns are smaller for both MSSA and MRSA grown on an AGA plate compared to on a standard TSA agar. This is mainly owing to the stress from antibiotics on the AGA plates. Compared to MSSA, MRSA scatter patterns are bigger, as observed in the case of 1-μg/mL AGA plates at 11 and 13 h. It has previously been reported that the magnitude of the colony slope determined the size of the scatter pattern, with steeper colony slopes showing bigger scatter patterns (Bae et al. 2010). This can be attributed to the growth of MRSA colonies in the vertical direction to resist the stress from oxacillin present in the AGA plate. The identification of differences in the scatter pattern between resistant and non-resistant strains is a complex task that cannot be accurately achieved by the naked eye. To better comprehend these differences, a machine-learning software has been designed to extract and compare features from the scatter pattern images. This process is elaborated in detail in Fig. 9.

### Revealing colony morphology using microscopy

**Fig. 8:**
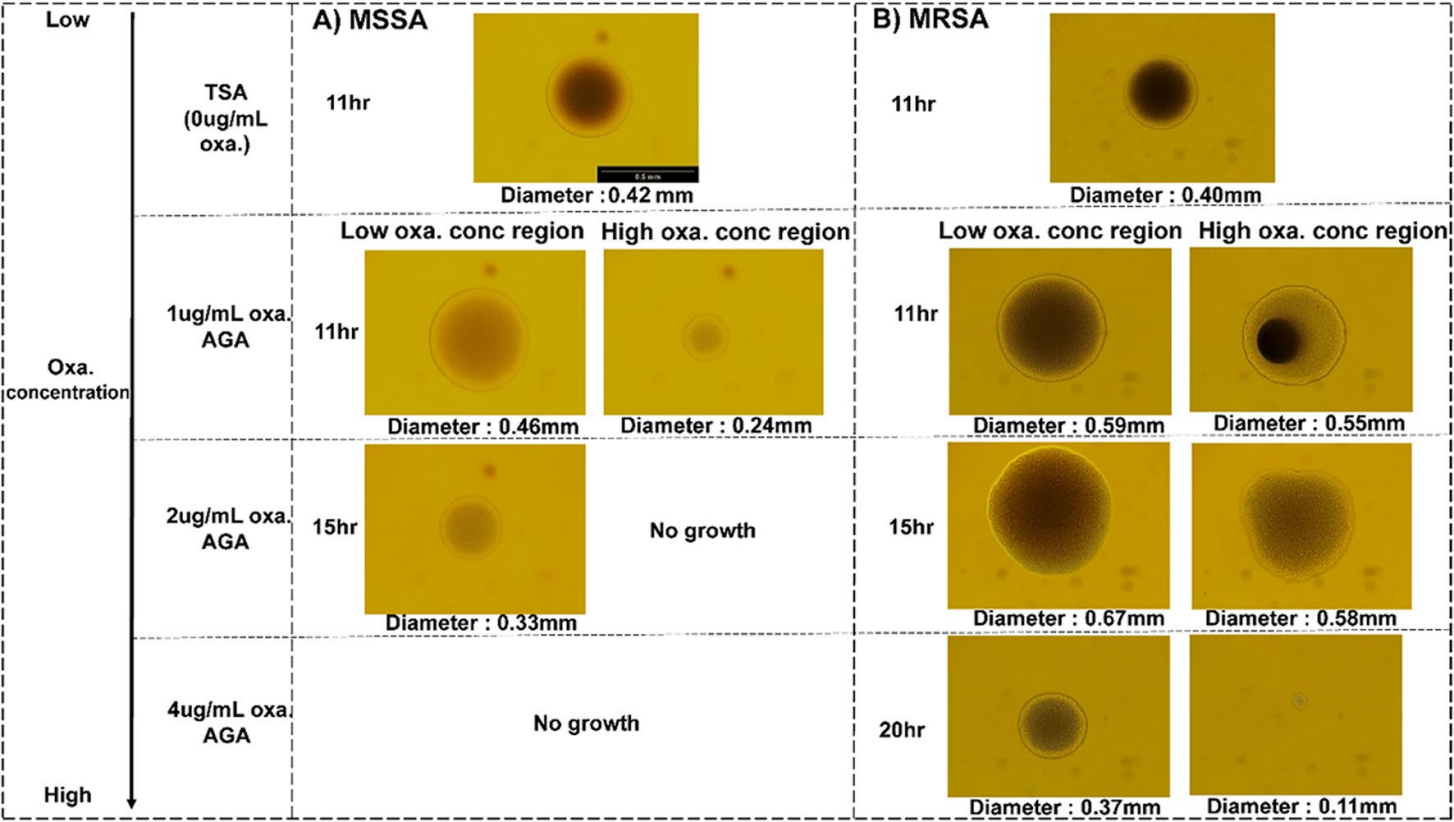
Microscopy images of MSSA and MRSA colonies grown on TSA and on AGA plates (1, 2, and 4 μg/mL). One example for each case is shown. The colony diameters were measured using ImageJ software. **A** One example of an MSSA colony (TSA agar positive control) and colonies from low and high oxacillin concentration regions are highlighted. MSSA colony size decreases (small colony variants) with increasing concentration of oxacillin. No growth of MSSA was observed on 4-μg/mL AGA plates and at high concentration region on 2-μg/mL AGA plates. **B** MRSA colonies were observed on all the AGA plates at both high and low concentration regions. However, heterogeneity in the colony morphology was observed. Colonies on high concentration regions of 1- and 2-μg/mL AGA plates showed different densities and colony shapes. At a high concentration of oxacillin, the size of the MRSA colony was substantially reduced, as observed at a high oxa concentration region of 4-μg/mL AGA plates (diameter 0.11 mm)

MSSA and MRSA colonies grown on AGA plates were examined under a bright-field microscope; the images captured to illustrate the colony heterogeneity observed in the scatter patterns are shown in Fig. 7. Magnification of × 10 was chosen, and the images of the colonies are shown in Fig. 8. MSSA and MRSA colonies grown on TSA agar were used as positive controls and were compared with colonies grown on 1-, 2-, and 4-μg/mL oxa AGA plates. For each AGA plate, one example of a colony at low and high concentrations of oxacillin is highlighted for both MSSA and MRSA (Fig. 8A and 8B). Using ImageJ software, the diameters of each colony were measured from microscope images. It is well known that *Staph. aureus* colonies are circular in shape when grown on agar in the absence of antibiotics. On TSA agar, the colony diameters were on average ± std deviation: 0.42 ± 0.04 mm and 0.4 ± 0.05 mm for MSSA and MRSA, respectively, after 11-h incubation. MSSA colony size was small (diameter 0.24 mm) at the high oxa concentration region of 1-μg/mL AGA plates compared to the low concentration region (diameter 0.46 mm) after 11-h incubation. The colony size of MSSA at the low oxa concentration region of 2-μg/mL AGA plates was also smaller in size (diameter 0.33 mm) compared to the colony at a low concentration region of 1-μg/mL AGA plates. As previously reported, no growth of MSSA was observed at high oxa concentration on 2-μg/mL AGA and at any region on 4-μg/mL AGA plates. In the case of MRSA colonies, interesting variation in colony morphology was observed based on the oxa concentration. For instance, the density at the center of an MRSA colony was observed to be skewed at a high oxa concentration region for 1 μg/mL AGA plate, but the diameter size remained very similar (diameter 0.55 mm) to the colony at low-concentration region (diameter 0.59 mm). Furthermore, irregular MRSA colony shapes were observed at a high-oxa-concentration region on 2 μg/mL AGA plate (diameter 0.58 mm). The colonies of MRSA at low- and high-oxa-concentration regions of 4-μg/mL AGA plates had diameters of 0.37 mm and 0.1 1mm, respectively, after 14-h incubation. This confirms that in the presence of environmental stress, such as antibiotics, the growth rates of both MSSA and MRSA are affected. Bacteria prefer to grow in regions with low antibiotic presence on the AGA plate, which can explain the variation in colony morphologies. Separate studies from O’Neill et al. (2007), Archer et al. (2011), and McCarthy et al. (2015) showed differences in biofilm phenotype between polysaccharide intercellular adhesion (PIA)-dependent MSSA and PIA-independent MRSA. PIA-independent biofilm formation in MRSA is due to cell-wall protein and cDNA-mediated tight cell-to-cell adhesion. This can explain the dense dark center regions in MRSA compared to the lighter center in MSSA in Fig. 8.

### Classification using machine-learning software designed for ELS

**Fig. 9:**
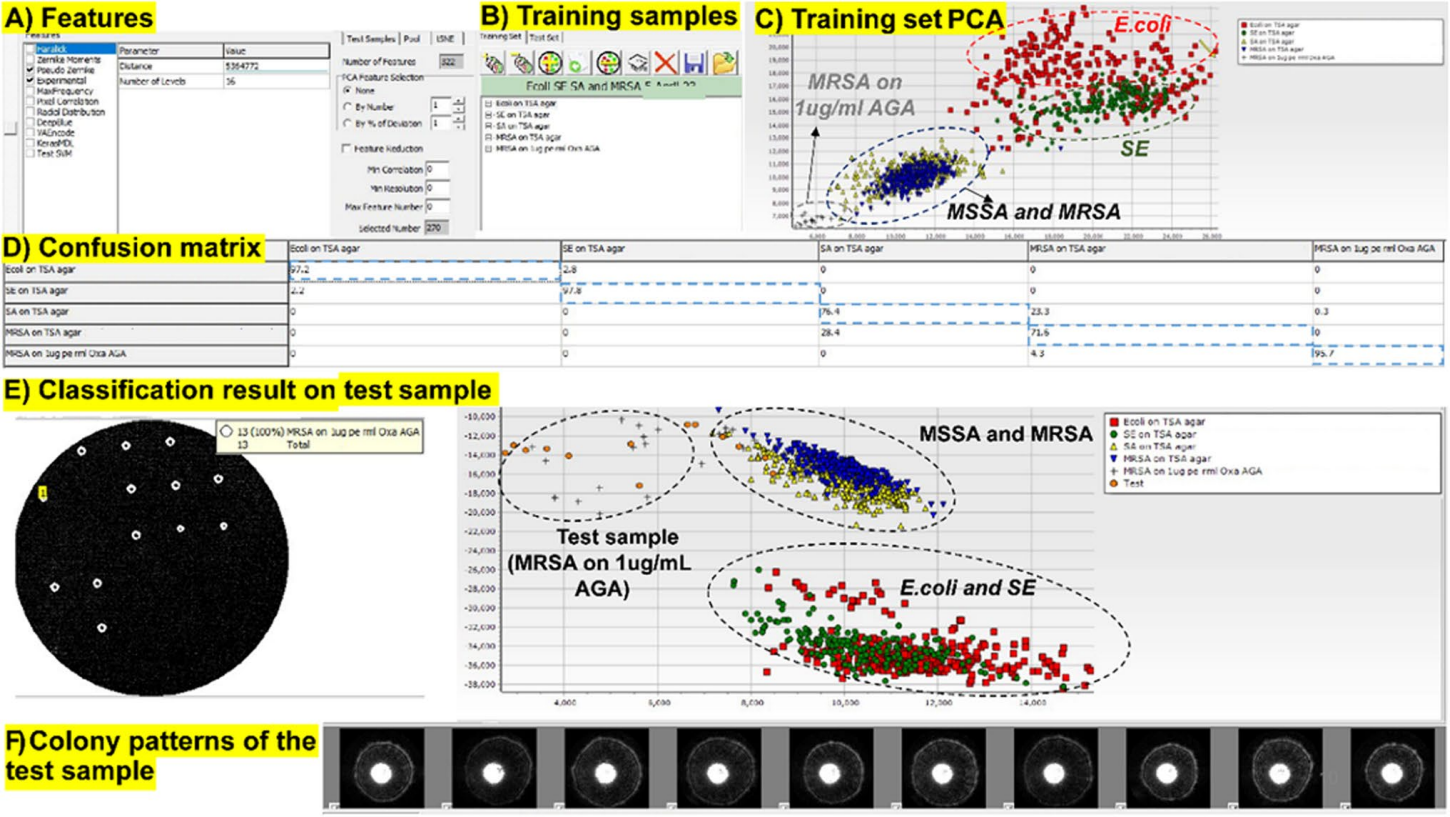
Screenshots showing the classification process of colony scatter patterns using machine-learning software. **A** Features selected in the software for classification. **B** Training samples from four different bacteria: *E. coli*, SE, MSSA, and MRSA grown for 11 h to create a ground-truth library. **C** Principal component analysis (PCA) performed on the training set showing clusters corresponding to bacterial classes. **D** Confusion matrix showing the summary of the model’s predictions. **E** A set of scatter patterns of MRSA colonies grown on 1-μg/mL oxa plates used as a test sample. Classification results show similarity of the test sample to the training scatter patterns of MRSA grown on 1-μg/mL oxa AGA plates. **F** Colony scatter-pattern image for each colony on the test-sample plate

In order to differentiate the scatter signatures of MSSA and MRSA on AGA plates (refer to Fig. 7), we employed a machine-learning approach. To conduct supervised classification, a scatter-pattern training set that encompasses all expected bacterial species must be established. This set serves as the ground-truth reference on which the classifier is trained to identify scatter patterns associated with unknown colonies. As previously mentioned, the image-analysis software extracts features, such as pseudo-Zernike moments, from the scatter patterns of the bacterial colonies, which are then utilized by the classifier in the recognition process. Figure 9 shows screenshots of the custom software used to perform the bacterial classification. A window offering different feature-extraction methods that are included in the software is shown in Fig. 9A.

Using the combination of feature families, a total of 322 features per scatter-pattern image were extracted. The software was initially trained with the colony scatter patterns taken after 11-h incubation from four different bacteria: *E. coli*, SE, MSSA, and MRSA grown on TSA agar along with two sets of MRSA colonies grown on 1-μg/mL oxa AGA plates as shown in Fig. 9B. Scatter patterns of these training samples were obtained on different days to account for natural biological variability. Table S1 in the supplementary data provides a summary of the number of colony scatter patterns and experimental repeats utilized for training the software for each bacterial species.

The result of principal component analysis (PCA) performed on the features extracted from the training set data showed that the *E. coli* and SE clusters can be clearly differentiated from SA and MRSA clusters, as shown in Fig. 9C. In addition, MRSA grown on 1-μg/mL oxa AGA shows a separate set of clusters outside the region of MSSA and MRSA clusters. Each point in the PCA plot corresponds to a single colony pattern.

The diagonal of the confusion matrix shows 97%, 98%, 76%, and 72% average recognition accuracy for *E. coli*, SE, MSSA (SA), and MRSA colonies grown on different days (Fig. 9D). For instance, the 97% accuracy for *E. coli* scatter patterns means that when *E. coli* test/sample scatter-pattern images are fed into the software and compared with the training set in the database (which has training scatter patterns of *E. coli*, *S. aureus*, *S. enteritis*, and MRSA), 97% of the time the model correctly identifies the test-set scatter pattern as belonging to *E. coli*. This shows that the model has been well trained and has high scatter-pattern prediction capability. About 96% prediction accuracy was observed for MRSA colonies grown on 1-μg/mL oxa AGA plates. It is noteworthy that there are 24% and 28% misclassification errors when distinguishing between MSSA and MRSA colonies.

As a proof of principle, one set of the MRSA colony patterns grown on 1-μg/mL oxa AGA plates was used as a test sample to check the classification performance of the software, as shown in Fig. 9E. The classification results show clustering of the test-set colony patterns to training MRSA scatter patterns grown on 1-μg/mL oxa plates. This was observed on the PCA plot. The software also shows individual scatter patterns of all the colonies in the test sample plate below the PCA plot (Fig. 9F).

## Discussion

The rapid spread of antibiotic resistance in bacteria has compromised current treatment methods and practices (Martinez and Baquero 2014). Hence, the methodology for bacteria classification and AMR detection continues to be of significant academic interest. Agar plating remains the preferred method for phenotypic analysis in pathogen detection owing to its high accuracy and reliability. This technique involves the screening and counting of media plates to obtain pure isolates, which can then be subject to further analysis. The use of agar plating has been widely adopted in both academic and industrial settings, owing to its ability to provide a clear and concise representation of the microbial population present in a given sample (Barton 2000; Galhano et al. 2021; Tang et al. 2017; Vasala et al. 2020) The agar-plating step is typically followed by the determination of the minimum inhibitory concentration (MIC), where the bacteria from positive cultures are grown again in the presence of antibiotics using methods such as disk diffusion, strips (E-test), or microbroth dilution (Baker et al. 1991; Balouiri et al. 2016; Kowalska-Krochmal and Dudek-Wicher 2021). The protocol commonly employed for bacterial growth on agar plates is the streak or spread plate technique, which necessitates multiple dilution steps to achieve single, isolated colonies. However, this approach not only extends the timeframe of the process but may also introduce false positives, stemming from an absence of colony growth at lower dilutions. If the concentration of bacteria is very high, colonies may overlap, making it difficult to isolate and analyze individual colonies downstream (Sanders 2012). The plating duration required to identify the bacteria varies from 2 to 7 days, depending on the type of initial sample. (Ferone et al. 2020; Zhang et al. 2019). This is mainly because agar plating is time consuming and requires multiple labor-intensive laboratory steps (Ferone et al. 2020; Terrones-Fernandez et al. 2023).

Genomic analysis, such as one involving PCR, is an often-preferred approach for identifying bacteria present in collected samples (Yamamoto 2002). Multiple studies have reported the detection of antibiotic resistance in bacteria from food and clinical samples (Anjum et al. 2017; Schwartz et al. 2003; Seedy et al. 2017; Sigmund et al. 2020). However, the performance of PCR depends on the purity of the starting sample and the inherent properties in food samples like beef can inhibit PCR assays (Galhano et al. 2021; Taylor et al. 2005). Moreover, PCR cannot differentiate between live and dead cells, as DNA is amplified from all cells (Adzitey et al. 2013; Narayana Iyengar et al. 2021; Sails et al. 1998).

Recently, spectrometry-based phenotypic identification methods, such as MALDI-TOF, have gained acceptance for rapid bacterial-identification tasks. This technique is sometimes combined with downstream genomic analysis, making it a sound academic approach (Nakano et al. 2014; Singhal et al. 2015; Vasala et al. 2020; Wang et al. 2014). MALDI-TOF is also used for performing antibiotic susceptibility testing (AST) (Vasala et al. 2020). However, bacterial identification and AST cannot be performed simultaneously, and it takes around 2 to 4 h to obtain AST (Vasala et al. 2020). Further, MALDI-TOF requires the technician to select the colony to be evaluated, allowing potential error and bias. The targeted colony is destroyed, making further analysis impossible.

There are various interesting alternative approaches, such as single-cell Raman or Raman-deuterium isotope labeling, which are rapid, non-destructive, and can be utilized for bacterial identification and AST. However, the signal intensity obtained from single-cell Raman spectroscopy is very weak, which demands further signal-enhancing techniques (Li et al. 2012; Rebrosova et al. 2022). In order to enhance the signal, surface-enhanced Raman spectroscopy (SERS) is employed for microbiological applications (Rebrosova et al. 2022; Samek et al. 2021). SERS provides an enhanced signal, but the result still depends upon many parameters such as media type, sample preparation method, and substrate-to-individual bacterial cell interactions, among others (Rebrosova et al. 2022; Samek et al. 2021; Witkowska et al. 2020). On the other hand, the Raman-deuterium isotope labeling can evaluate bacterial metabolism and determine MIC via this route. However, most of the studies provide information on the sensitivity of bacteria to antibiotics (sensitive vs. non-sensitive) but still cannot determine the exact MIC (Zhang et al. 2022). All these techniques also require growing bacteria for up to 24 h before analysis can be performed (Rebrosova et al. 2022; Zhang et al. 2022). In addition, similar to our technique and other phenotypic approaches, the spectroscopy-based systems rely on databases of identified bacterial strains (Rebrosova et al. 2022). Further, because of national sample processing needs, isotope labeling has become a technology less attractive to most laboratories.

In order to tackle many of the challenges, we present a new approach for rapid identification and categorization of bacteria with the goal of enhancing AMR detection. This is accomplished by utilizing a cell sorter, whose capability to precisely position individual bacterial cells onto designated regions of an agar plate we have demonstrated, without requiring any dilution techniques.

Cell sorters have the ability to sort individual cells with high throughput and efficiency. Moreover, modern cell sorters are capable of accurately positioning the sorted cells, making them a valuable tool for automated single-cell plating (Ambriz-Avina et al. 2014; Robinson et al. 2023). Latest flow cytometers such as Bigfoot are capable of processing high bacterial concentrations in the sample, up to 70,000 events per second. They allow instantaneous observation of the presence or absence of bacteria in the sample through light-scatter plots (FSC vs. SSC plots). By optimizing the flow rate, event rate, cell concentration, and gating area on the scatter plot, it is possible to reliably and accurately capture single bacterial cells per droplet up to the limitations governed by Poissonian statistics.

In the context of handling pathogenic bacteria, it is standard practice to manage the samples within a biosafety level 2 (BSL-2) hood (Burnett et al. 2009). Therefore, in order to sort pathogenic organisms using FC-based cell sorters, the instrument must be kept inside a BSL-2 hood (Robinson et al. 2023; Schmid et al. 2007). One of the primary difficulties is that a significant number of instruments are not compatible with biosafety hoods. Even when an instrument does fit, the user’s ability to operate it with ease may be compromised (Robinson et al. 2023). This problem is addressed in our work by utilizing a Bigfoot cell sorter that is equipped with a BSL-2 biosafety system and is capable of handling pathogenic organisms such as MRSA in normal laboratory settings with ease. To our knowledge, this represents the first application of a traditional cell sorter (i.e., not relying on microfluidic technology) for the purpose of depositing single cells of highly pathogenic organisms to cultivate well-defined colonies. Flow cytometers are already used in clinical settings (mostly for immune-cell detection and counting), so our protocol may be integrated into existing procedures.

Sorting and depositing single organisms leading to the formation of bacterial colonies on AGA plates has significant advantages. Traditionally, after collecting the sample, bacteria are grown via spread-plate technique, and plates are incubated for at least 24 to 48 h. From these positive cultures, the colonies are again cultured in the presence of antibiotics to determine MIC, which means an additional 24 to 48 h. By directly depositing bacteria on the AGA plate, multiple culturing steps can be avoided, and the time required to identify the presence of antibiotic-resistant strains in the sample can be decreased, and a much quicker “yes/no” result can be obtained. Growing bacteria directly on an AGA plate can reduce the time of antibiotic-resistant bacterial identification from a total of 48–72 h (“gold standard” agar plating) to 11–24 h, depending on the starting sample type. In addition, since plating is still one of the recognized standard methods that is widely used, our approach can be easily integrated into an existing protocol in hospital settings. By observing the growth of bacteria in a high antibiotic concentration area on a 2-μg/mL or any region on a 4-μg/mL oxacillin AGA plate, the presence of antimicrobial-resistant strains like MRSA can be readily confirmed. This can significantly reduce diagnosis time, which is especially important in cases of severe infectious diseases linked to sepsis. Also, in contrast to a PCR-based analysis pipeline, with a cell sorter it is feasible to incorporate a bacterial staining step (such as a combination of SYTO9 and propidium iodide) prior to sorting to verify the existence of viable and non-viable microorganisms. As demonstrated in our research, the combination of cell sorter-based patterning on AGA plates with machine-learning-based ELS technology enables the identification and classification of bacterial species based on their distinct colony scatter patterns. This method is rapid, non-destructive, label-free, and can provide answers in a couple of minutes by comparing the unknown bacterial colony pattern to the patterns recorded in the database. This advancement holds significant importance in the management of polymicrobial-resistant samples and high-density bacterial samples, particularly those derived from patients afflicted with urinary tract infections or from contaminated food and water sources.

Finally, by combining a flow cytometer, AGA plate, and ELS technology, the time taken from sample collection to result can be significantly reduced by at least half of the time required by standard agar plating methods. This is achieved by reducing the number of plating steps and by utilizing the ELS identification techniques. Various alternative deposition patterns, such as spot arrangements for 384-well plates and 1536-well plates, can be utilized in our analytical technique. Cells can also be placed on rectangular plates. Using this approach can greatly decrease the required number of plates and minimize the overall expenses, as multiple colonies can be grown on a single-agar plate. Supplementary Fig. S4A displays the arrangement of two groups of *E. coli* on a TSA agar plate utilizing a 384-spot map. This configuration allows for a maximum of 208 colonies to be accommodated on a circular plate. Supplementary Fig. S4B displays the growth of *Salmonella enteritis* (SE) on a rectangular XLT4 agar plate after 24 h of incubation arranged in a 384-spot map. We are currently implementing the 1536-well plate layouts to enable colonies to be in closer proximity and to observe interactions with adjacent colonies. By arranging two distinct bacterial species in neighboring locations, we would be able to analyze their interaction and monitor the growth patterns of the bacteria. At present, we are in the initial phases of creating the necessary ground-truth database for the ELS procedure. Our primary aim is to record the scatter patterns of colonies formed by the microorganisms that are responsible for the most prevalent infections, as well as their respective resistant strains. To enhance the accuracy of classifying and identifying bacteria from tested samples, we intend to integrate advanced techniques such as feature learning into our data-processing pipeline.

## Supplementary Information

Below is the link to the electronic supplementary material.

Supplementary file1 (PDF 3558 KB)

## Data Availability

All data supporting the findings of this study are presented within the paper and the supplementary documents. The raw data, such as flow cytometry files (FCS), and the scatter patterns are available upon request.

## References

[CR1] Abdou Mohamed MA, Kozlowski HN, Kim J, Zagorovsky K, Kantor M, Feld JJ, Mubareka S, Mazzulli T, Chan WCW (2021) Diagnosing antibiotic resistance using nucleic acid enzymes and gold nanoparticles. ACS Nano 15(6):9379–9390. 10.1021/acsnano.0c09902 33970612 10.1021/acsnano.0c09902

[CR2] Adzitey F, Huda N, Ali GR (2013) Molecular techniques for detecting and typing of bacteria, advantages and application to foodborne pathogens isolated from ducks. 3 Biotech 3(2):97–107. 10.1007/s13205-012-0074-4 28324565 10.1007/s13205-012-0074-4 PMC3597138

[CR3] Alipour F, Ahmadi M, Javadi S (2014) Evaluation of different methods to detect methicillin resistance in *Staphylococcus aureus* (MRSA). J Infect Public Health 7(3):186–191. 10.1016/j.jiph.2014.01.007 24656721 10.1016/j.jiph.2014.01.007

[CR4] Alsulami TS, Zhu XY, Abdelhaseib MU, Singh AK, Bhunia AK (2018) Rapid detection and differentiation of *Staphylococcus* colonies using an optical scattering technology. Anal Bioanal Chem 410(22):5445–5454. 10.1007/s00216-018-1133-4 29796901 10.1007/s00216-018-1133-4

[CR5] Ambriz-Avina V, Contreras-Garduno JA, Pedraza-Reyes M (2014) Applications of flow cytometry to characterize bacterial physiological responses. Biomed Res Int 2014:461941. 10.1155/2014/461941 25276788 10.1155/2014/461941 PMC4174974

[CR6] Anjum MF, Zankari E, Hasman H (2017) Molecular methods for detection of antimicrobial resistance. Microbiol Spectr 5(6). 10.1128/microbiolspec.ARBA-0011-2017 10.1128/microbiolspec.arba-0011-2017 PMC11687549 29219107

[CR7] Archer NK, Mazaitis MJ, Costerton JW, Leid JG, Powers ME, Shirtliff ME (2011) *Staphylococcus aureus* biofilms: properties, regulation, and roles in human disease. Virulence 2(5):445–459. 10.4161/viru.2.5.17724 21921685 10.4161/viru.2.5.17724 PMC3322633

[CR8] Bae E, Bai N, Aroonnual A, Bhunia AK, Hirleman ED (2011) Label-free identification of bacterial microcolonies via elastic scattering. Biotechnol Bioeng 108(3):637–644. 10.1002/bit.22980 21246511 10.1002/bit.22980

[CR9] Bae E, Patsekin V, Rajwa B, Bhunia AK, Holdman C, Davisson VJ, Hirleman ED, Robinson JP (2012a) Development of a microbial high-throughput screening instrument based on elastic light scatter patterns. Rev Sci Instrum 83(4):044304. 10.1063/1.3697853 22559555 10.1063/1.3697853 PMC3339897

[CR10] Bae E, Ying D, Kramer D, Patsekin V, Rajwa B, Holdman C, Sturgis J, Davisson VJ, Robinson JP (2012b) Portable bacterial identification system based on elastic light scatter patterns. J Biol Eng 6(1):12. 10.1186/1754-1611-6-12 22929757 10.1186/1754-1611-6-12 PMC3490744

[CR11] Bae E, Kim H, Rajwa B, Thomas JG, Robinson JP (2016) Current status and future prospects of using advanced computer-based methods to study bacterial colonial morphology. Expert Rev Anti Infect Ther 14(2):207–218. 10.1586/14787210.2016.1122524 26582139 10.1586/14787210.2016.1122524

[CR12] Bae E, Bai N, Aroonnual A, Robinson JP, Bhunia AK, Hirleman ED (2010) Modeling light propagation through bacterial colonies and its correlation with forward scattering patterns. J Biomed Opt 15(4). 10.1117/1.3463003 10.1117/1.3463003 20799796

[CR13] Bagwell CB, Adams EG (1993) Fluorescence spectral overlap compensation for any number of flow cytometry parameters. Ann N Y Acad Sci 677(1):167–184. 10.1111/j.1749-6632.1993.tb38775.x 8494206 10.1111/j.1749-6632.1993.tb38775.x

[CR14] Baker CN, Stocker SA, Culver DH, Thornsberry C (1991) Comparison of the E test to agar dilution, broth microdilution, and agar diffusion susceptibility testing techniques by using a special challenge set of bacteria. J Clin Microbiol 29(3):533–538. 10.1128/jcm.29.3.533-538.1991 2037671 10.1128/jcm.29.3.533-538.1991 PMC269813

[CR15] Balouiri M, Sadiki M, Ibnsouda SK (2016) Methods for in vitro evaluating antimicrobial activity: a review. J Pharm Anal 6(2):71–79. 10.1016/j.jpha.2015.11.005 29403965 10.1016/j.jpha.2015.11.005 PMC5762448

[CR16] Baltekin O, Boucharin A, Tano E, Andersson DI, Elf J (2017) Antibiotic susceptibility testing in less than 30 min using direct single-cell imaging. P Natl Acad Sci USA 114(34):9170–9175. 10.1073/pnas.1708558114 10.1073/pnas.1708558114 PMC5576829 28790187

[CR17] Banada PP, Guo S, Bayraktar B, Bae E, Rajwa B, Robinson JP, Hirleman ED, Bhunia AK (2007) Optical forward-scattering for detection of Listeria monocytogenes and other Listeria species. Biosens Bioelectron 22(8):1664–1671. 10.1016/j.bios.2006.07.028 16949268 10.1016/j.bios.2006.07.028

[CR18] Banada PP, Huff K, Bae E, Rajwa B, Aroonnual A, Bayraktar B, Adil A, Robinson JP, Hirleman ED, Bhunia AK (2009) Label-free detection of multiple bacterial pathogens using light-scattering sensor. Biosens Bioelectron 24(6):1685–1692. 10.1016/j.bios.2008.08.053 18945607 10.1016/j.bios.2008.08.053

[CR19] Barton MD (2000) Antibiotic use in animal feed and its impact on human healt. Nutr Res Rev 13(2):279–299. 10.1079/095442200108729106 19087443 10.1079/095442200108729106

[CR20] Bayraktar B, Banada PP, Hirleman ED, Bhunia AK, Robinson JP, Rajwa B (2006) Feature extraction from light-scatter patterns of Listeria colonies for identification and classification. J Biomed Opt 11(3):34006. 10.1117/1.2203987 16822056 10.1117/1.2203987

[CR21] Bryson V, Szybalski W (1952) Microbial selection. Science 116(3003):45–51. 10.1126/science.116.3003.45 14950177

[CR22] Burnett LC, Lunn G, Coico R (2009) Biosafety: guidelines for working with pathogenic and infectious microorganisms. Curr Protoc Microbiol Chapter 1(1). 10.1002/9780471729259.mc01a01s13 (Unit 1A 1) 10.1002/9780471729259.mc01a01s13 PMC7162325 19412909

[CR23] Buzalewicz I, Suchwałko A, Trzciński P, Sas-Paszt L, Sumorok B, Kowal K, Kozera R, Wieliczko A, Podbielska H (2019) Integrated multi-channel optical system for bacteria characterization and its potential use for monitoring of environmental bacteria. Biomed Opt Express 10(3):1165–1183. 10.1364/BOE.10.001165 30891337 10.1364/BOE.10.001165 PMC6420290

[CR24] Cangelosi GA, Meschke JS (2014) Dead or alive: molecular assessment of microbial viability. Appl Environ Microbiol 80(19):5884–5891. 10.1128/AEM.01763-14 25038100 10.1128/AEM.01763-14 PMC4178667

[CR25] CDC (2019) Methicillin-resistant *Staphylococcus aureus* (MRSA)- Laboratory testing. PUblisher. https://www.cdc.gov/mrsa/lab/index.html

[CR26] Cherkaoui A, Hibbs J, Emonet S, Tangomo M, Girard M, Francois P, Schrenzel J (2010) Comparison of two matrix-assisted laser desorption ionization-time of flight mass spectrometry methods with conventional phenotypic identification for routine identification of bacteria to the species level. J Clin Microbiol 48(4):1169–1175. 10.1128/JCM.01881-09 20164271 10.1128/JCM.01881-09 PMC2849558

[CR27] Chong CW, Raveendran P, Mukundan R (2003) An efficient algorithm for fast computation of pseudo-zernike moments. Int J Pattern Recogn 17(6):1011–1023. 10.1142/S0218001403002769

[CR28] CLSI (2020) Performance standards for antimicrobial susceptibility testing M100. 30 edn CLSI, p 402. https://clsi.org/standards/products/microbiology/documents/m100/?gad_source=1&gclid=CjwKCAjw5ImwBhBtEiwAFHDZx5UgJvByxZcYpbUkJCU_cNZKiGcTnj9zgi0CWTQpUUQkrfACyUdwaxoCjSoQAvD_BwE

[CR29] Davey HM, Kell DB (1996) Flow cytometry and cell sorting of heterogeneous microbial populations: the importance of single-cell analyses. Microbiol Rev 60(4):641–696. 10.1128/mr.60.4.641-696.1996 8987359 10.1128/mr.60.4.641-696.1996 PMC239459

[CR30] Dietvorst J, Ferrer-Vilanova A, Iyengar SN, Russom A, Vigues N, Mas J, Vilaplana L, Marco MP, Guirado G, Munoz-Berbel X (2022) Bacteria detection at a single-cell Level through a cyanotype-based photochemical reaction. Anal Chem 94(2):787–792. 10.1021/acs.analchem.1c03326 34931815 10.1021/acs.analchem.1c03326 PMC8771638

[CR31] Ferone M, Gowen A, Fanning S, Scannell AGM (2020) Microbial detection and identification methods: Bench top assays to omics approaches. Compr Rev Food Sci Food Saf 19(6):3106–3129. 10.1111/1541-4337.12618 33337061 10.1111/1541-4337.12618

[CR32] Flusser J, Suk T, Zitová B (2016) 2D and 3D image analysis by moments. John Wiley & Sons, Ltd 320–397. 10.1002/9781119039402

[CR33] Fomda BA, Thokar MA, Khan A, Bhat JA, Zahoor D, Bashir G, Majid A, Ray P (2014) Nasal carriage of Methicillin-resistant *Staphylococcus aureus* among healthy population of Kashmir India. Indian J Med Microbiol 32(1):39–43. 10.4103/0255-0857.124296 24399386 10.4103/0255-0857.124296

[CR34] Gajic I, Kabic J, Kekic D, Jovicevic M, Milenkovic M, Culafic DM, Trudic A, Ranin L, Opavski N (2022) Antimicrobial susceptibility testing: A comprehensive review of currently used methods. Antibiotics-Basel 11(4). 10.3390/antibiotics11040427 10.3390/antibiotics11040427 PMC9024665 35453179

[CR35] Galhano S, Ferrari G, Panzenhagen P, de Jesus ACS, Conte-Junior CA (2021) Antimicrobial resistance gene detection methods for bacteria in animal-based foods: A brief review of highlights and advantages. Microorganisms 9(5):923. 10.3390/microorganisms9050923 33925810 10.3390/microorganisms9050923 PMC8146338

[CR36] Gosiewski T, Jurkiewicz-Badacz D, Sroka A, Brzychczy-Wloch M, Bulanda M (2014) A novel, nested, multiplex, real-time PCR for detection of bacteria and fungi in blood. BMC Microbiol 14:144. 10.1186/1471-2180-14-144 24893651 10.1186/1471-2180-14-144 PMC4049433

[CR37] Grégori G, Patsekin V, Rajwa B, Jones J, Ragheb K, Holdman C, Robinson JP (2012) Hyperspectral cytometry at the single-cell level using a 32-channel photodetector. Cytometry A 81A(1):35–44. 10.1002/cyto.a.21120 10.1002/cyto.a.21120 22173900

[CR38] Grégori G, Rajwa B, Patsekin V, Jones J, Furuki M, Yamamoto M, Paul Robinson J (2014) Hyperspectral cytometry. In: Fienberg HG, Nolan GP (eds) High-Dimensional Single Cell Analysis: Mass Cytometry, Multi-parametric Flow Cytometry and Bioinformatic Techniques. Springer, Berlin Heidelberg, Berlin, Heidelberg, pp 191–210

[CR39] Gurung RR, Maharjan P, Chhetri GG (2020) Antibiotic resistance pattern of *Staphylococcus aureus* with reference to MRSA isolates from pediatric patients. Future Sci OA 6(4):FSO464. 10.2144/fsoa-2019-0122 32257376 10.2144/fsoa-2019-0122 PMC7117559

[CR40] Harbarth S, Hawkey PM, Tenover F, Stefani S, Pantosti A, Struelens MJ (2011) Update on screening and clinical diagnosis of meticillin-resistant *Staphylococcus aureus* (MRSA). Int J Antimicrob Agents 37(2):110–117. 10.1016/j.ijantimicag.2010.10.022 21163628 10.1016/j.ijantimicag.2010.10.022

[CR41] Hartman BJ, Tomasz A (1984) Low-affinity penicillin-binding protein associated with beta-lactam resistance in *Staphylococcus aureus*. J Bacteriol 158(2):513–516. 10.1128/jb.158.2.513-516.1984 6563036 10.1128/jb.158.2.513-516.1984 PMC215458

[CR42] Hibbing ME, Fuqua C, Parsek MR, Peterson SB (2010) Bacterial competition: surviving and thriving in the microbial jungle. Nat Rev Microbiol 8(1):15–25. 10.1038/nrmicro2259 19946288 10.1038/nrmicro2259 PMC2879262

[CR43] Huff K, Aroonnual A, Littlejohn AEF, Rajwa B, Bae E, Banada PP, Patsekin V, Hirleman ED, Robinson JP, Richards GP, Bhunia AK (2012) Light-scattering sensor for real-time identification of *Vibrio parahaemolyticus*, *Vibrio vulnificus* and *Vibrio cholerae* colonies on solid agar plate. Microb Biotechnol 5(5):607–620. 10.1111/j.1751-7915.2012.00349.x 22613192 10.1111/j.1751-7915.2012.00349.x PMC3815873

[CR44] Huff K, Banada P, Bae E, Bayraktar B, Rajwa B, Robinson J, Hirleman E, Richards G, Bhunia AK (2006) Detection and identification of foodborne pathogens to genus and species levels using a noninvasive modified light scatterometer - bardot. Am Soc Microbiol 075. https://www.ars.usda.gov/research/publications/publication/?seqNo115=191624

[CR45] Irvine A, Dang T, Dundar MM, Rajwa B (2022) IM: An R-package for computation of image moments and moment invariants. arXiv 2210.16485 [cs.CV]. 10.48550/arXiv.2210.16485

[CR46] Iyengar SN, Kumar T, Martensson G, Russom A (2021) High resolution and rapid separation of bacteria from blood using elasto-inertial microfluidics. Electrophor 42(23):2538–2551. 10.1002/elps.202100140 10.1002/elps.202100140 34510466

[CR47] Iyengar SN (2021) Novel microfluidic based sample preparation methods for rapid separation and detection of viable bacteria from blood for sepsis diagnostics. KTH-Royal institute of technology. https://kth.diva-portal.org/smash/record.jsf?pid=diva2%3A1610140&dswid=3702

[CR48] Jolliffe IT, Cadima J (2016) Principal component analysis: a review and recent developments. Philos Trans A Math Phys Eng Sci 374(2065):20150202. 10.1098/rsta.2015.0202 26953178 10.1098/rsta.2015.0202 PMC4792409

[CR49] Kennedy D, Wilkinson MG (2017) Application of flow cytometry to the detection of pathogenic bacteria. Curr Issues Mol Biol 23:21–38. 10.21775/cimb.023.021 28561007 10.21775/cimb.023.021

[CR50] Kennedy D, Cronin UP, Wilkinson MG (2011) Responses of *Escherichia coli*, *Listeria monocytogenes*, and *Staphylococcus aureus* to simulated food processing treatments, determined using fluorescence-activated cell sorting and plate counting. Appl Environ Microbiol 77(13):4657–4668. 10.1128/AEM.00323-11 21602370 10.1128/AEM.00323-11 PMC3127720

[CR51] Khotanzad A, Hong YH (1990) Invariant image recognition by Zernike moments. IEEE Trans Pattern Anal Mach Intell 12(5):489–497. 10.1109/34.55109

[CR52] Kim WY, Kim YS (2000) A region-based shape descriptor using Zernike moments. Signal Process Image Commun 16(1–2):95–102. 10.1016/S0923-5965(00)00019-9

[CR53] Kotilainen P, Jalava J, Meurman O, Lehtonen OP, Rintala E, Seppala OP, Eerola E, Nikkari S (1998) Diagnosis of meningococcal meningitis by broad-range bacterial PCR with cerebrospinal fluid. J Clin Microbiol 36(8):2205–2209. 10.1128/JCM.36.8.2205-2209.1998 9665992 10.1128/jcm.36.8.2205-2209.1998 PMC105011

[CR54] Kowalska-Krochmal B, Dudek-Wicher R (2021) The minimum inhibitory concentration of antibiotics: methods, interpretation, clinical relevance. Pathogens 10(2). 10.3390/pathogens10020165 10.3390/pathogens10020165 PMC7913839 33557078

[CR55] Krishnamurthy V, Saha A, Renushri BV, Nagaraj ER (2014) Methicillin resistant *Staphylococcus aureus* carriage, antibiotic resistance and molecular pathogenicity among healthy individuals exposed and not exposed to hospital environment. J Clin Diagn Res 8(7):DC04-8. 10.7860/JCDR/2014/8409.4638 25177563 10.7860/JCDR/2014/8409.4638 PMC4149069

[CR56] Kumar T, Ramachandraiah H, Iyengar SN, Banerjee I, Martensson G, Russom A (2021) High throughput viscoelastic particle focusing and separation in spiral microchannels. Sci Rep 11(1):8467. 10.1038/s41598-021-88047-4 33875755 10.1038/s41598-021-88047-4 PMC8055915

[CR57] Kunze N, Moerer O, Steinmetz N, Schulze MH, Quintel M, Perl T (2015) Point-of-care multiplex PCR promises short turnaround times for microbial testing in hospital-acquired pneumonia–an observational pilot study in critical ill patients. Ann Clin Microbiol Antimicrob 14:33. 10.1186/s12941-015-0091-3 26071191 10.1186/s12941-015-0091-3 PMC4469099

[CR58] Lever J, Krzywinski M, Atman N (2017) Points of significance principal component analysis. Nat Methods 14(7):641–642. 10.1038/nmeth.4346

[CR59] Li M, Xu J, Romero-Gonzalez M, Banwart SA, Huang WE (2012) Single cell Raman spectroscopy for cell sorting and imaging. Curr Opin Biotechnol 23(1):56–63. 10.1016/j.copbio.2011.11.019 22138495 10.1016/j.copbio.2011.11.019

[CR60] Li YL, Fernandez R, Duran I, Molina-Lopez RA, Darwich L (2021) Antimicrobial resistance in bacteria isolated from cats and dogs from the Iberian Peninsula. Front Microbiol 11. 10.3389/fmicb.2020.621597 10.3389/fmicb.2020.621597 PMC7874003 33584590

[CR61] Liu MF, He YX, Ye B (2007) Image Zernike moments shape feature evaluation based on image reconstruction. Geo-Spat Inf Sci 10(3):191–195. 10.1007/s11806-007-0060-x

[CR62] Liu Y, Li J, Du J, Hu M, Bai H, Qi J, Gao C, Wei T, Su H, Jin J, Gao P (2011) Accurate assessment of antibiotic susceptibility and screening resistant strains of a bacterial population by linear gradient plate. Sci China Life Sci 54(10):953–960. 10.1007/s11427-011-4230-6 22038008 10.1007/s11427-011-4230-6

[CR63] Llor C, Bjerrum L (2014) Antimicrobial resistance: risk associated with antibiotic overuse and initiatives to reduce the problem. Ther Adv Drug Saf 5(6):229–241. 10.1177/2042098614554919 25436105 10.1177/2042098614554919 PMC4232501

[CR64] Lowy FD (1998) *Staphylococcus aureus* infections. N Engl J Med 339(8):520–532. 10.1056/NEJM199808203390806 9709046 10.1056/NEJM199808203390806

[CR65] Lowy FD (2003) Antimicrobial resistance: the example of *Staphylococcus aureus*. J Clin Invest 111(9):1265–1273. 10.1172/JCI18535 12727914 10.1172/JCI18535 PMC154455

[CR66] Lukkana M, Jantrakajorn S, Wongtavatchai J (2016) Antimicrobial susceptibility and enrofloxacin resistance of *streptococcal* bacteria from farmed Nile tilapia, Oreochromis niloticus (Linnaeus 1758) in Thailand. Aquac Res 47(10):3136–3144. 10.1111/are.12764

[CR67] Marcoux PR, Dupoy M, Cuer A, Kodja J-L, Lefebvre A, Licari F, Louvet R, Narassiguin A, Mallard F (2014) Optical forward-scattering for identification of bacteria within microcolonies. Appl Microbiol Biotechnol 98(5):2243–2254. 10.1007/s00253-013-5495-4 24413976 10.1007/s00253-013-5495-4

[CR68] Martin MJ, Thottathil SE, Newman TB (2015) Antibiotics overuse in animal agriculture: A call to action for health care providers. Am J Public Health 105(12):2409–2410. 10.2105/Ajph.2015.302870 26469675 10.2105/AJPH.2015.302870 PMC4638249

[CR69] Martinez JL, Baquero F (2014) Emergence and spread of antibiotic resistance: setting a parameter space. Ups J Med Sci 119(2):68–77. 10.3109/03009734.2014.901444 24678768 10.3109/03009734.2014.901444 PMC4034563

[CR70] McCarthy H, Rudkin JK, Black NS, Gallagher L, O’Neill E, O’Gara JP (2015) Methicillin resistance and the biofilm phenotype in *Staphylococcus aureus*. Front Microbiol 5. 10.3389/fcimb.2015.00001 10.3389/fcimb.2015.00001 PMC4309206 25674541

[CR71] Migenda N, Möller R, Schenck W (2024) Adaptive local principal component analysis improves the clustering of high-dimensional data. Pattern Recognit 146:110030. 10.1016/j.patcog.2023.110030. (ARTN)

[CR72] Mukundan R, Ramakrishnan KR (1995) Fast computation of legendre and zernike moments. Pattern Recognit 28(9):1433–1442. 10.1007/s11554-007-0058-5

[CR73] Nakano S, Matsumura Y, Kato K, Yunoki T, Hotta G, Noguchi T, Yamamoto M, Nagao M, Ito Y, Takakura S, Ichiyama S (2014) Differentiation of vanA-positive Enterococcus faecium from vanA-negative E. faecium by matrix-assisted laser desorption/ionisation time-of-flight mass spectrometry. Int J Antimicrob Agents 44(3):256–259. 10.1016/j.ijantimicag.2014.05.006 25104134 10.1016/j.ijantimicag.2014.05.006

[CR74] Narayana Iyengar S, Dietvorst J, Ferrer-Vilanova A, Guirado G, Munoz-Berbel X, Russom A (2021) Toward rapid detection of viable bacteria in whole blood for early sepsis diagnostics and susceptibility testing. ACS Sens 6(9):3357–3366. 10.1021/acssensors.1c01219 34410700 10.1021/acssensors.1c01219 PMC8477386

[CR75] O’Neill E, Pozzi C, Houston P, Smyth D, Humphreys H, Robinson DA, O’Gara JP (2007) Association between methicillin susceptibility and biofilm regulation in *Staphylococcus aureus* isolates from device-related infections. J Clin Microbiol 45(5):1379–1388. 10.1128/JCM.02280-06 17329452 10.1128/JCM.02280-06 PMC1865887

[CR76] Otto M (2010) Basis of virulence in community-associated methicillin-resistant *Staphylococcus aureus*. Annu Rev Microbiol 64:143–162. 10.1146/annurev.micro.112408.134309 20825344 10.1146/annurev.micro.112408.134309

[CR77] Pan W, Zhao J, Chen Q (2015) Classification of foodborne pathogens using near infrared (NIR) laser scatter imaging system with multivariate calibration. Sci Rep 5(1):9524. 10.1038/srep09524 25860918 10.1038/srep09524 PMC5381752

[CR78] Piras C, Soggiu A, Greco V, Martino PA, Del Chierico F, Putignani L, Urbani A, Nally JE, Bonizzi L, Roncada P (2015) Mechanisms of antibiotic resistance to enrofloxacin in uropathogenic *Escherichia coli* in dog. J Proteomics 127:365–376. 10.1016/j.jprot.2015.05.040 26066767 10.1016/j.jprot.2015.05.040

[CR79] Rajwa B, Dundar MM, Akova F, Bettasso A, Patsekin V, Dan Hirleman E, Bhunia AK, Robinson JP (2010) Discovering the unknown: detection of emerging pathogens using a label-free light-scattering system. Cytom Part A 77A(12):1103–1112. 10.1002/cyto.a.20978 10.1002/cyto.a.20978 PMC3224816 21108360

[CR80] Rebrosova K, Samek O, Kizovsky M, Bernatova S, Hola V, Ruzicka F (2022) Raman spectroscopy-a novel method for identification and characterization of microbes on a single-cell level in clinical settings. Front Cell Infect Microbiol 12:866463. 10.3389/fcimb.2022.866463 35531343 10.3389/fcimb.2022.866463 PMC9072635

[CR81] Robinson JP, Rajwa B, Grégori G, Jones J, Patsekin V (2004) Collection hardware for high speed multispectral single particle analysis. Cytometry 59A:12–12

[CR82] Robinson JP, Ostafe R, Iyengar SN, Rajwa B, Fischer R (2023) Flow Cytometry: the next Revolution. Cells 12(14):1875. 10.3390/cells12141875 37508539 10.3390/cells12141875 PMC10378642

[CR83] Robinson JP (2004) Multispectral cytometry: the next generation. Biophotonics International, Laurin Publishing, Pittsfield, MA, USA October, p 36–40. https://www.researchgate.net/publication/318865510_Multispectral_Cytometry_The_next_Generation#fullTextFileContent

[CR84] Roederer M (2001) Spectral compensation for flow cytometry: visualization artifacts, limitations, and caveats. Cytometry 45(3):194–205 11746088 10.1002/1097-0320(20011101)45:3<194::aid-cyto1163>3.0.co;2-c

[CR85] Rychert J (2019) Benefits and limitations of MALDI-TOF mass spectrometry for the identification of microorganisms. J Infectiology Epidemiol 1–5. 10.29245/2689-9981/2019/4.1142

[CR86] Sails AD, Bolton FJ, Fox AJ, Wareing DR, Greenway DL (1998) A reverse transcriptase polymerase chain reaction assay for the detection of thermophilic Campylobacter spp. Mol Cell Probes 12(5):317–322. 10.1006/mcpr.1998.0184 9778457 10.1006/mcpr.1998.0184

[CR87] Salam MA, Al-Amin MY, Pawar JS, Akhter N, Lucy IB (2023) Conventional methods and future trends in antimicrobial susceptibility testing. Saudi J Biol Sci 30(3):103582. 10.1016/j.sjbs.2023.103582 36852413 10.1016/j.sjbs.2023.103582 PMC9958398

[CR88] Samek O, Bernatová S, Dohnal F (2021) The potential of SERS as an AST methodology in clinical settings. Nanophotonics 10(10):2537–2561. 10.1515/nanoph-2021-0095

[CR89] Sanders ER (2012) Aseptic laboratory techniques: plating methods. J Vis Exp 63:e3064. 10.3791/3064 10.3791/3064 PMC4846335 22617405

[CR90] Schmid I, Lambert C, Ambrozak D, Perfetto SP (2007) Standard safety practices for sorting of unfixed cells. Curr Protoc Cytom Chapter 3(Unit3):6. 10.1002/0471142956.cy0306s39 10.1002/0471142956.cy0306s39 18770851

[CR91] Schwartz T, Kohnen W, Jansen B, Obst U (2003) Detection of antibiotic-resistant bacteria and their resistance genes in wastewater, surface water, and drinking water biofilms. FEMS Microbiol Ecol 43(3):325–335. 10.1111/j.1574-6941.2003.tb01073.x 19719664 10.1111/j.1574-6941.2003.tb01073.x

[CR92] Seedy FRE, Samy AA, Salam HSH, Khairy EA, Koraney AA (2017) Polymerase chain reaction detection of genes responsible for multiple antibiotic resistance Staphylococcus aureus isolated from food of animal origin in Egypt. Vet World 10(10):1205–1211. 10.14202/vetworld.2017.1205-1211 29184366 10.14202/vetworld.2017.1205-1211 PMC5682265

[CR93] Shapiro HM (2003) Practical flow cytometry, 3rd edn. John Wiley & Sons, Inc. 10.1002/0471722731

[CR94] Sigmund IK, Renz N, Feihl S, Morgenstern C, Cabric S, Trampuz A (2020) Value of multiplex PCR for detection of antimicrobial resistance in samples retrieved from patients with orthopaedic infections. BMC Microbiol 20(1):88. 10.1186/s12866-020-01741-7 32290833 10.1186/s12866-020-01741-7 PMC7155317

[CR95] Singh AK, Sun X, Bai X, Kim H, Abdalhaseib MU, Bae E, Bhunia AK (2015) Label-free, non-invasive light scattering sensor for rapid screening of Bacillus colonies. J Microbiol Methods 109:56–66. 10.1016/j.mimet.2014.12.012 25533215 10.1016/j.mimet.2014.12.012

[CR96] Singh AK, Bettasso AM, Bae E, Rajwa B, Dundar MM, Forster MD, Liu LX, Barrett B, Lovchik J, Robinson JP, Hirleman ED, Bhunia AK (2014) Laser Optical Sensor, a label-free on-plate Salmonella enterica colony detection tool. Mbio 5(1). 10.1128/mBio.01019-13 10.1128/mBio.01019-13 PMC3950520 24496794

[CR97] Singhal N, Kumar M, Kanaujia PK, Virdi JS (2015) MALDI-TOF mass spectrometry: an emerging technology for microbial identification and diagnosis. Front Microbiol 6:791. 10.3389/fmicb.2015.00791 26300860 10.3389/fmicb.2015.00791 PMC4525378

[CR98] Sinha M, Jupe J, Mack H, Coleman TP, Lawrence SM, Fraley SI (2018) Emerging technologies for molecular diagnosis of sepsis. Clin Microbiol Rev 31(2). 10.1128/CMR.00089-17 10.1128/CMR.00089-17 PMC5967692 29490932

[CR99] Steen HB (1990) Light scattering measurement in an arc lamp-based flow cytometer. Cytometry A 11(2):223–230. 10.1002/cyto.990110202 10.1002/cyto.990110202 2180652

[CR100] Tahamtan A, Ardebili A (2020) Real-time RT-PCR in COVID-19 detection: issues affecting the results. Expert Rev Mol Diagn 20(5):453–454. 10.1080/14737159.2020.1757437 32297805 10.1080/14737159.2020.1757437 PMC7189409

[CR101] Tang Y, Kim H, Singh AK, Aroonnual A, Bae E, Rajwa B, Fratamico PM, Bhunia AK (2014) Light scattering sensor for direct identification of colonies of *Escherichia coli* serogroups O26, O45, O103, O111, O121, O145 and O157. PLoS ONE 9(8):e105272. 10.1371/journal.pone.0105272 25136836 10.1371/journal.pone.0105272 PMC4138183

[CR102] Tang KL, Caffrey NP, Nobrega DB, Cork SC, Ronksley PE, Barkema HW, Polachek AJ, Ganshorn H, Sharma N, Kellner JD, Ghali WA (2017) Restricting the use of antibiotics in food-producing animals and its associations with antibiotic resistance in food-producing animals and human beings: a systematic review and meta-analysis. Lancet Planet Health 1(8):e316–e327. 10.1016/S2542-5196(17)30141-9 29387833 10.1016/S2542-5196(17)30141-9 PMC5785333

[CR103] Taylor TM, Elhanafi D, Drake M, Jaykus LA (2005) Effect of food matrix and cell growth on PCR-based detection of Escherichia coli O157:H7 in ground beef. J Food Prot 68(2):225–232. 10.4315/0362-028x-68.2.225 15726961 10.4315/0362-028x-68.2.225

[CR104] Teague MR (1980) Image-analysis via the general-theory of moments. J Opt Soc Am 70(8):920–930. 10.1364/Josa.70.000920

[CR105] Teh CH, Chin RT (1988) On image-analysis by the methods of moments. Ieee T Pattern Anal 10(4):496–513. 10.1109/34.3913

[CR106] Terrones-Fernandez I, Casino P, Lopez A, Peiro S, Rios S, Nardi-Ricart A, Garcia-Montoya E, Asensio D, Marques AM, Castilla R, Gamez-Montero PJ, Pique N (2023) Improvement of the pour plate method by separate sterilization of agar and other medium components and reduction of the agar concentration. Microbiol Spectr 11(1):e0316122. 10.1128/spectrum.03161-22 36625633 10.1128/spectrum.03161-22 PMC9927588

[CR107] ThermoFisher (2020) Bigfoot spectral cell sorter features. PUblisher. https://www.thermofisher.com/us/en/home/life-science/cell-analysis/flow-cytometry/flow-cytometers/bigfoot-spectral-cell-sorter/features.html

[CR108] Trung NT, Thau NS, Bang MH, Song LH (2019) PCR-based Sepsis@Quick test is superior in comparison with blood culture for identification of sepsis-causative pathogens. Sci Rep 9(1):13663. 10.1038/s41598-019-50150-y 31541157 10.1038/s41598-019-50150-y PMC6754458

[CR109] Vasala A, Hytonen VP, Laitinen OH (2020) Modern tools for rapid diagnostics of antimicrobial resistance. Front Cell Infect Microbiol 10:308. 10.3389/fcimb.2020.00308 32760676 10.3389/fcimb.2020.00308 PMC7373752

[CR110] Veal DA, Deere D, Ferrari B, Piper J, Attfield PV (2000) Fluorescence staining and flow cytometry for monitoring microbial cells. J Immunol Methods 243(1–2):191–210. 10.1016/s0022-1759(00)00234-9 10986415 10.1016/s0022-1759(00)00234-9

[CR111] Wang LJ, Lu XX, Wu W, Sui WJ, Zhang G (2014) Application of matrix-assisted laser desorption ionization time-of-flight mass spectrometry in the screening of vanA-positive Enterococcus faecium. Eur J Mass Spectrom (chichester) 20(6):461–465. 10.1255/ejms.1298 25905870 10.1255/ejms.1298

[CR112] WHO (2021) Antimicrobial resistance. https://www.who.int/news-room/fact-sheets/detail/antimicrobial-resistance

[CR113] Witkowska E, Nicinski K, Korsak D, Dominiak B, Waluk J, Kaminska A (2020) Nanoplasmonic sensor for foodborne pathogens detection. Towards development of ISO-SERS methodology for taxonomic affiliation of Campylobacter spp. J Biophotonics 13(5):e201960227. 10.1002/jbio.201960227 32022438 10.1002/jbio.201960227

[CR114] Xu J, Millar BC, Moore JE, Murphy K, Webb H, Fox AJ, Cafferkey M, Crowe MJ (2003) Employment of broad-range 16S rRNA PCR to detect aetiological agents of infection from clinical specimens in patients with acute meningitis–rapid separation of 16S rRNA PCR amplicons without the need for cloning. J Appl Microbiol 94(2):197–206. 10.1046/j.1365-2672.2003.01839.x 12534811 10.1046/j.1365-2672.2003.01839.x

[CR115] Yamamoto Y (2002) PCR in diagnosis of infection: detection of bacteria in cerebrospinal fluids. Clin Diagn Lab Immunol 9(3):508–514. 10.1128/cdli.9.3.508-514.2002 11986253 10.1128/CDLI.9.3.508-514.2002 PMC119969

[CR116] Yeung KY, Ruzzo WL (2001) Principal component analysis for clustering gene expression data. Bioinformatics 17(9):763–774. 10.1093/bioinformatics/17.9.763 11590094 10.1093/bioinformatics/17.9.763

[CR117] Yoon EJ, Jeong SH (2021) MALDI-TOF mass spectrometry technology as a tool for the rapid diagnosis of antimicrobial resistance in bacteria. Antibiotics (Basel) 10(8). 10.3390/antibiotics10080982 10.3390/antibiotics10080982 PMC8388893 34439032

[CR118] Zhang DX, Bi HY, Liu BH, Qao L (2018) Detection of pathogenic microorganisms by microfluidics based analytical methods. Anal Chem 90(9):5512–5520. 10.1021/acs.analchem.8b00399 29595252 10.1021/acs.analchem.8b00399

[CR119] Zhang Y, Hu A, Andini N, Yang S (2019) A ‘culture’ shift: Application of molecular techniques for diagnosing polymicrobial infections. Biotechnol Adv 37(3):476–490. 10.1016/j.biotechadv.2019.02.013 30797092 10.1016/j.biotechadv.2019.02.013 PMC6447436

[CR120] Zhang W, He S, Hong W, Wang P (2022) A review of Raman-based technologies for bacterial identification and antimicrobial susceptibility testing. Photonics 9(3). 10.3390/photonics9030133

