# Supplementary material for: Identifying antibiotic-resistant strains via cell sorting and elastic-light-scatter phenotyping
Source: Appl Microbiol Biotechnol. 2024 Jul 3;108:406. doi: 10.1007/s00253-024-13232-0 (PMC11222266; doi:10.1007/s00253-024-13232-0)
Supplement: Supplementary file 1 [file 253_2024_13232_MOESM1_ESM.pdf]

Applied Microbiology and Biotechnology

Supplementary data

Identifying Antibiotic-Resistant Strains via Cell Sorting and Elastic-Light-Scatter Phenotyping

Sharath Narayana Iyengar[1], Brianna Dowden[1], Kathy Ragheb[1], Valery Patsekin[1], Bartek Rajwa[4], Euiwon Bae[3], J Paul Robinson[1,2*]

1 Department of Basic Medical Sciences, Purdue University, West Lafayette, IN 47907, USA

2 Weldon School of Biomedical Engineering, Purdue University, West Lafayette, IN 47907, USA

3 School of Mechanical Engineering, Purdue University, West Lafayette, IN 47907, USA

4 Bindley Bioscience Center, Purdue University, West Lafayette, IN 47907, USA

*Corresponding author; Email ID: wombat@purdue.edu, Phone: +1765-494-6449

Determination of minimum inhibitory concentration (MIC) using broth microdilution

Using 96 well plate, the minimum inhibitory concentration for both MSSA and MRSA was determined as shown in Fig.S1. Six oxacillin concentrations (1,2,4,16,32, and 64ug/mL) were prepared, and equal concentrations of bacteria were added to all concentrations (Fig.S1A for MSSA and S1C for MRSA). TSA broth without oxacillin antibiotics was used as a positive control and TSA broth in the absence of bacteria and antibiotics was used as a negative control. Three sets for each concentration were performed and alternative wells were left blank to prevent cross contamination. For each of the cases, optical density (OD) was measured after 24hr of incubation at 600nm. Graph in Fig.S1B and S1D shows the OD measured for MSSA and MRSA for different concentrations of oxacillin. It can be observed that there was no significant growth of MSSA in all the antibiotic concentrations, whereas MRSA growth was observed until 32ug/mL oxacillin. For MRSA the MIC is >=64ug/mL oxacillin.

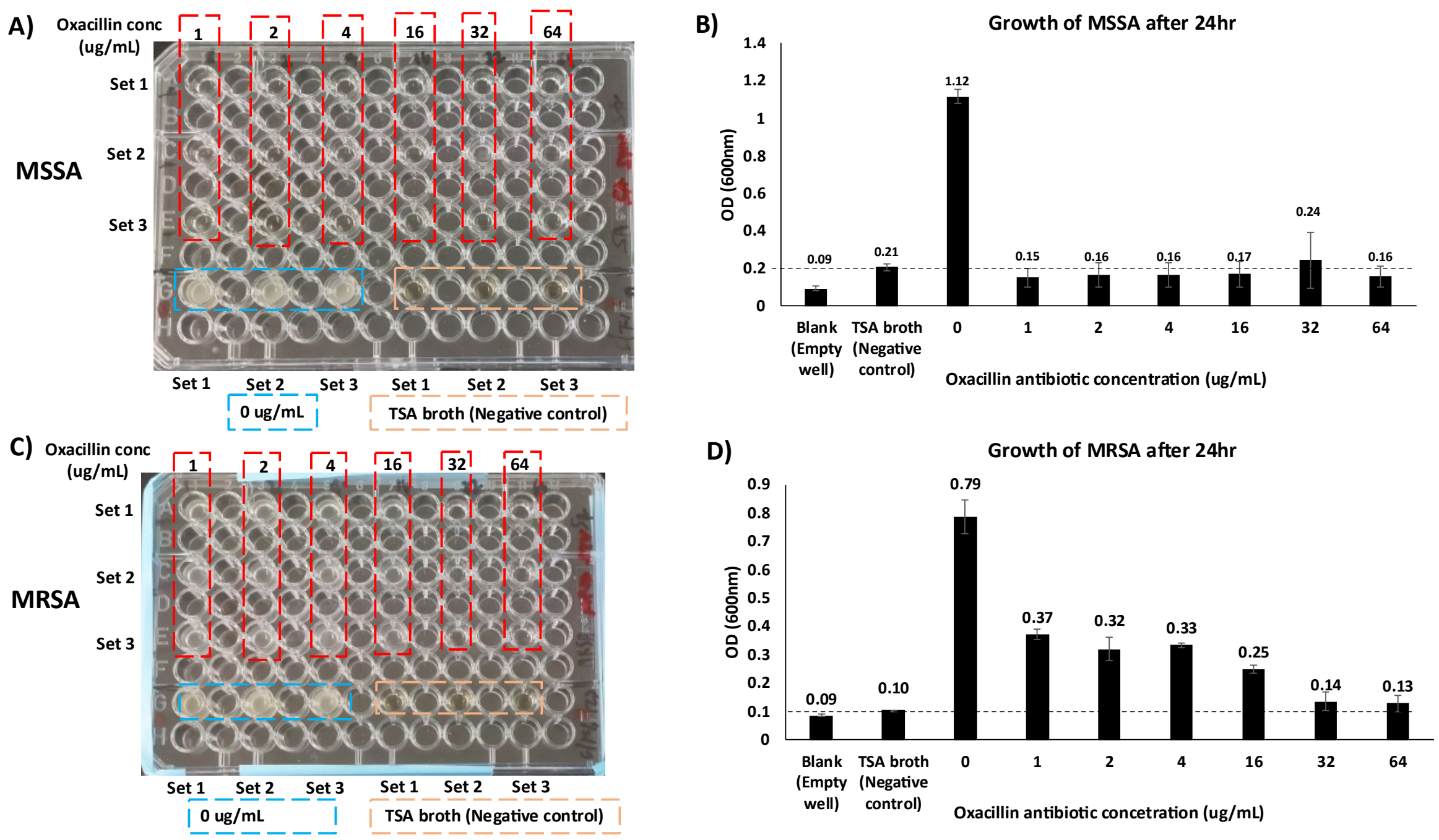

Fig.S1. MIC determination using broth microdilution technique. (A) and (C) Growth of MSSA and MRSA on 96 well plate in different concentration of oxacillin is shown (n=3). Tryptic soya broth (TSB) and TSB without antibiotics were used as negative and positive controls respectively.(B) and (D) Graph showing the OD (600nm) measured for each antibiotic concentration after 24hr of incubation. No growth of MSSA was observed in any oxacillin concentration (based on the negative control threshold line) whereas the MIC for MRSA was found to be >=64ug/mL oxacillin concentration.

Details of number of colonies performed over different days of experiments for feature extraction and bacterial classification

The below table shows different bacterial species, total number of colonies per bacterial species and the number of experiments per each bacterium that were used

for training the ELS software and for performing bacterial classification and identification (Fig.9). For instance, in the case of *E.coli*, 284 colonies grown on TSA agar for 11hr from 4 days of experiments were used to extract the scatter pattern features for the purpose of training and classification.

SL.No	Bacteria name	Total colonies used for training set	Number of experiments (in days)	Incubation time (hr)	Agar type
1.	*E.coli*	284	4	11	TSA
2.	SE	184	3	11	TSA
3.	MSSA	384	7	11	TSA
4.	MRSA (REAR-16)	300	5	11	TSA
5.	MRSA on 1ug/mL AGA	23	2	11	TSA

Table S1. Different bacteria classes and other parameters used for colony scatter pattern extraction and classification.

Preparation of AGA plate using oxacillin antibiotics

Preparation of AGA plate is described in material and methods section 5.3. The steps involved for preparing two layers for AGA plate are shown in schematic of Fig.S2A. A small black mat with 3mm thickness for slating the agar plate while preparing the first layer of agar on AGA plate is shown in Fig.S2B.

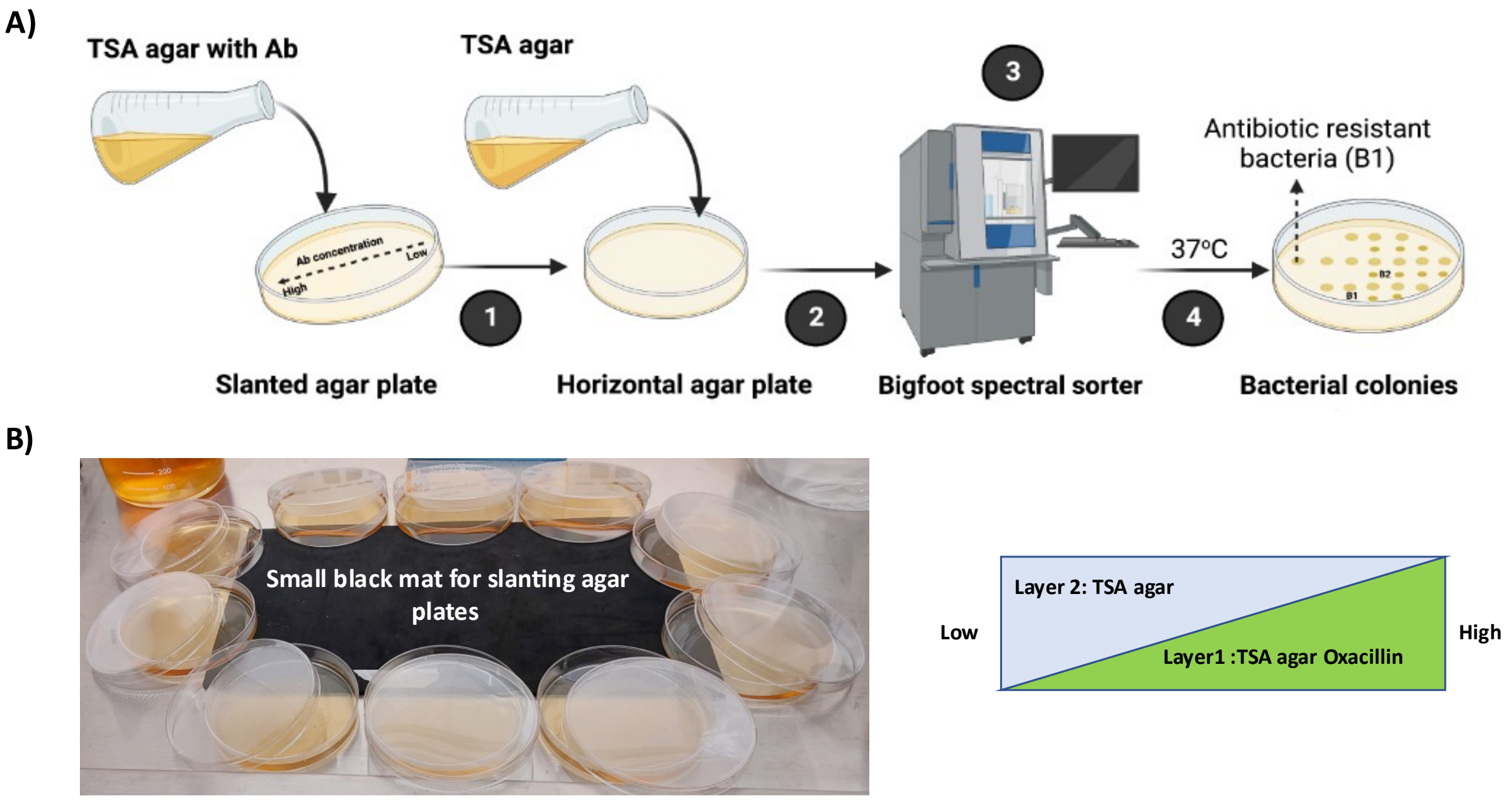

Fig.S2. Preparation of AGA plate. (A) Schematic showing the steps involved in preparing AGA plate and patterning bacteria using AGA plate. (B) Setup used for preparing the slanted first layer of agar on AGA plate using a small black mat is shown.

Growth of MSSA and MRSA on 4 and 16ug/mL AGA plate

MSSA and MRSA were grown on 4 and 16ug/mL oxa AGA plate as described in Fig.5A and 5B after patterning using Bigfoot. After 15hr of incubation, no MSSA or MRSA colonies were found growing on any region of the AGA plate. However, growth of MRSA was observed at low oxa concentration region on 4ug/mL at 31hr of incubation as shown in Fig,5C. No growth of MSSA or MRSA was observed on 16ug/mL even after 31hr of incubation (not shown).

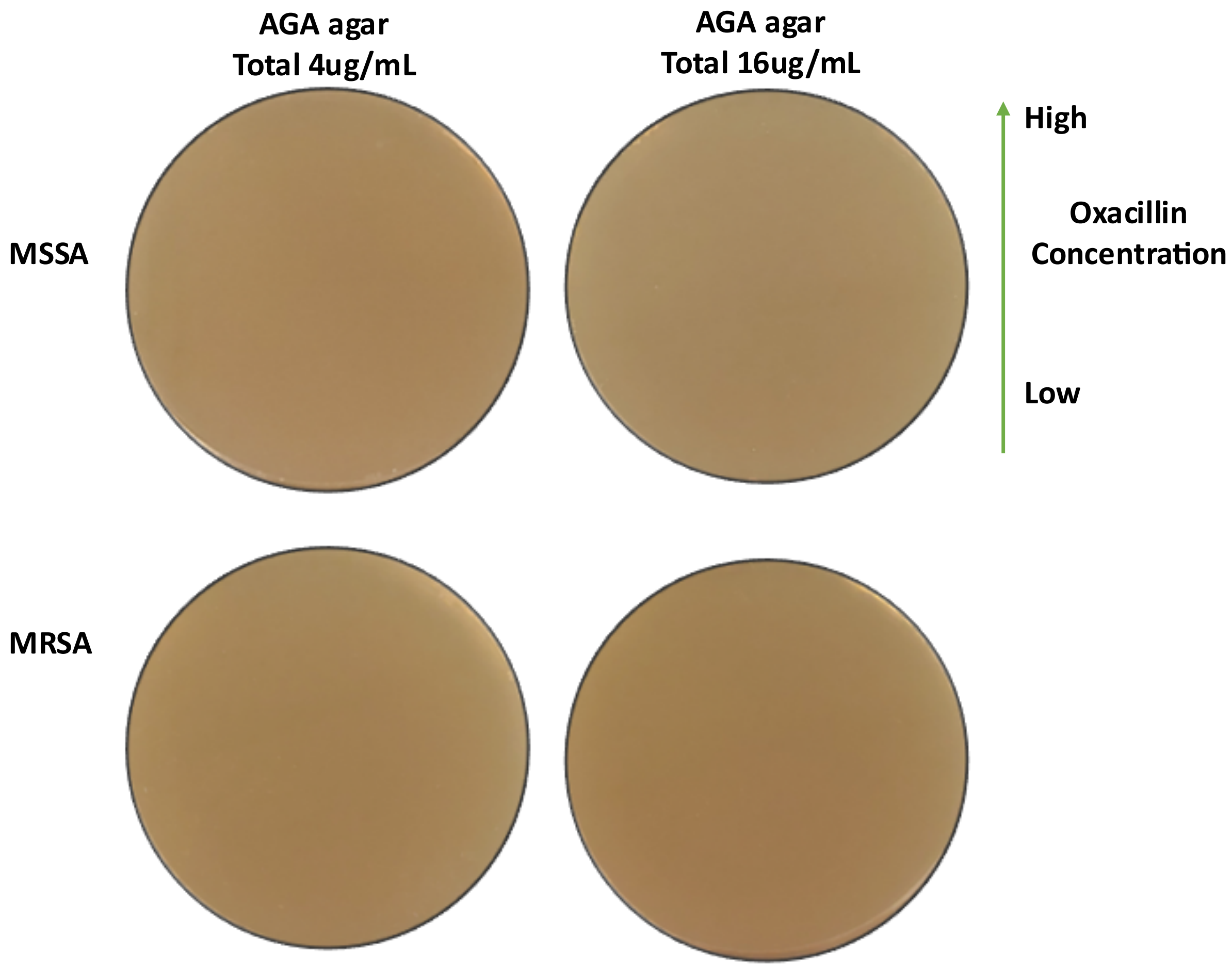

Fig.S3. Growth of MSSA and MRSA on 4 and 16ug/mL AGA plate. MSSA and MRSA did not grow on any region of the 4 and 16ug/mL AGA plate until 15hr of incubation.

Bright filed microscope videos of bacteria comparing it to ELS patterns

The colonies that were patterned using Bigfoot on the agar plate were visualized and focused using a bright field microscope. Video examples of *E.coli* and *Staph.aureus* colony on the agar plate can be obtained in the below DOI links (Figshare):

E.coli- doi:10.6084/m9.figshare.24534706

Staph.aureus- doi:10.6084/m9.figshare.24534709

The colonies were initially focused so that the outer layer of the colony is clearly visible. The focus was changed by moving the objective away from the agar plate (along the

height) and the video was recorded. An example of the scatter pattern of each of *E.coli* and *Staph.aureus* taken from the ELS device is shown. Interestingly, the colony scatter pattern from the ELS and the colony pattern observed by changing the focus using a bright field microscope show similar patterns. This shows that the growth of bacterial colonies along the height of the colony has a greater influence on the scatter pattern in addition to the colony morphology along the width.

Patterning of bacteria using 384 well plate map using Bigfoot

Circular TSA agar plate was used to pattern *E.coli* using 384 well plate map in bigfoot. Fig.S4A show *E.coli* colonies formed on the TSA agar plate after 11hr of incubation. To increase the colony numbers per plate, rectangular agar plates can be used as an alternative to circular plates. Fig.S4B shows growth of SE after 24hr of incubation which was patterned on rectangular XLT4 agar plate using a 384 well plate map in bigfoot.

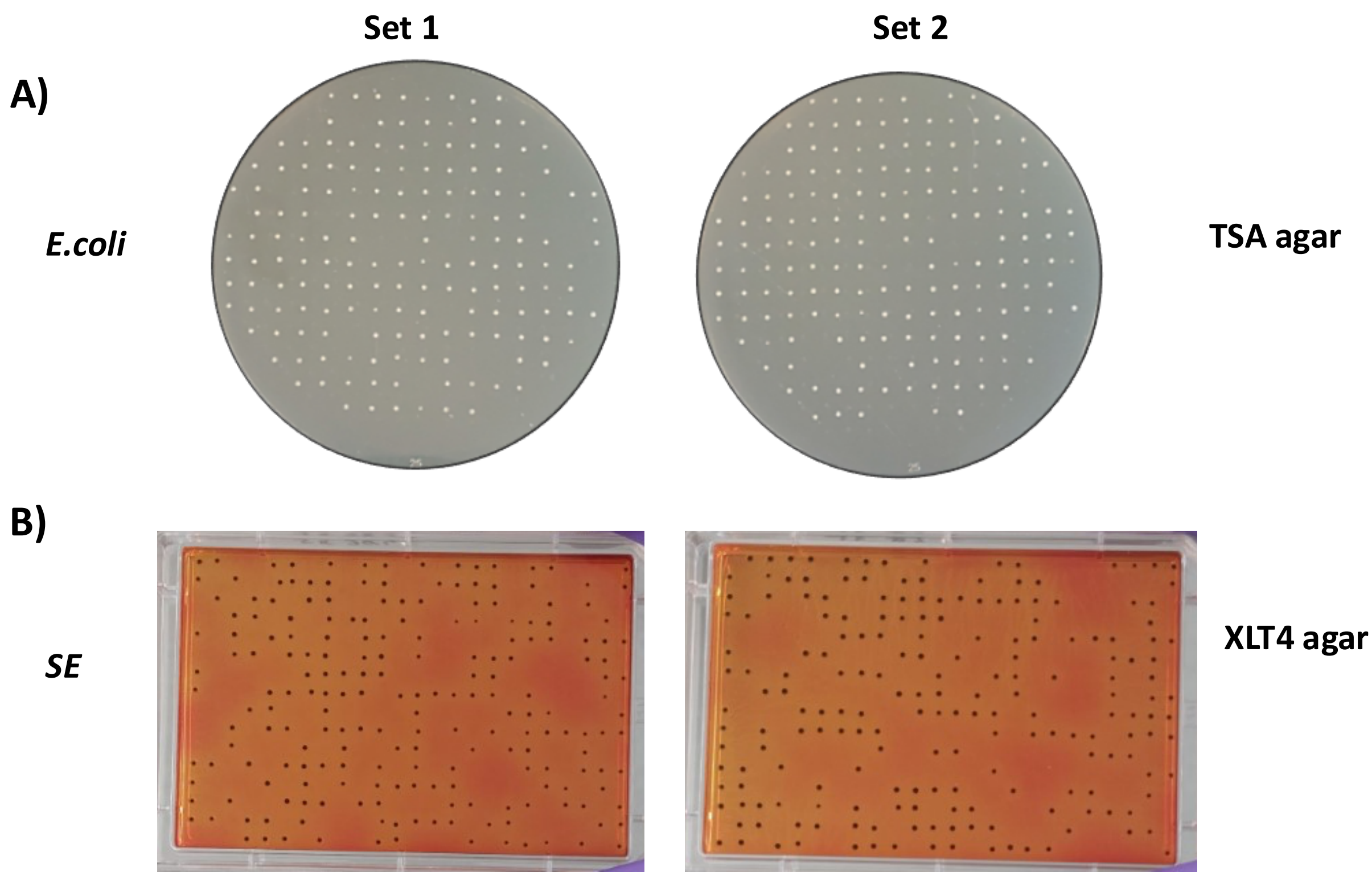

Fig.S4. Patterning bacteria on agar plate using 384 well plate map using Bigfoot. (A) Two sets of *E.coli* colonies grown on TSA agar plate after 11hr of incubation which was patterned using the 384 well plate map is shown. (B) Growth of SE on a rectangular agar plate using 384 well plate map after 24hr of incubation.
